# Unearthing who and Y at Harewood Cemetery and inference of George Washington’s Y-chromosomal haplotype

**DOI:** 10.1016/j.isci.2024.109353

**Published:** 2024-03-28

**Authors:** Courtney Cavagnino, Göran Runfeldt, Michael Sager, Roberta Estes, Andreas Tillmar, Ellen M. Greytak, Jacqueline Tyler Thomas, Elise Anderson, Jennifer Daniels-Higginbotham, Katelyn Kjelland, Kimberly Sturk-Andreaggi, Thomas J. Parsons, Timothy P. McMahon, Charla Marshall

**Affiliations:** 1 Armed Forces Medical Examiner System’s Armed Forces DNA Identification Laboratory (AFMES-AFDIL), Dover Air Force Base, DE 19902, USA; 2 SNA International, LLC, Alexandria, VA 22314, USA; 3 FamilyTreeDNA, Gene by Gene, Houston, TX 77008, USA; 4 Department of Forensic Genetics and Forensic Toxicology, National Board of Forensic Medicine, 587 58 Linköping, Sweden; 5 Department of Biomedical and Clinical Sciences, Faculty of Medicine and Health Sciences, Linköping University, 582 25 Linköping, Sweden; 6 Parabon NanoLabs, Inc., Reston, VA 20190, USA; 7 Amentum Services Inc., Germantown, MD 20876, USA; 8 Smithsonian Institution, National Museum of Natural History, Washington DC 20560, USA; 9 Forensic Science Program, The Pennsylvania State University, University Park, PA 1680116802, USA

**Keywords:** Bioinformatics, Human genetics

## Abstract

An excavation conducted at Harewood Cemetery to identify the unmarked grave of Samuel Washington resulted in the discovery of burials presumably belonging to George Washington’s paternal grandnephews and their mother, Lucy Payne. To confirm their identities this study examined Y-chromosomal, mitochondrial, and autosomal DNA from the burials and a living Washington descendant. The burial’s Y-STR profile was compared to FamilyTreeDNA’s database, which resulted in a one-step difference from the living descendant and an exact match to another Washington. A more complete Y-STR and Y-SNP profile from the descendant was inferred to be the Washington Y profile. Kinship comparisons performed in relation to the descendant, who is a 4^th^ and 5^th^ degree relative of the putative individuals, resulted in >37,000 overlapping autosomal SNPs and strong statistical support with likelihood ratios exceeding one billion. This study highlights the benefits of a multi-marker approach for kinship prediction and DNA-assisted identification of historical remains.

## Highlights

• Washington burials from mid-1800s predicted to be a mother and two full siblings
• Full siblings individualized using auSNPs of living “double Washington” descendant
• Y-STR profile of the male burial consistent with living Washingtons in FTDNA
• The Y-DNA profile of George Washington’s patriline belongs to haplogroup R-BY32422

## Graphical abstract

**Figure:**
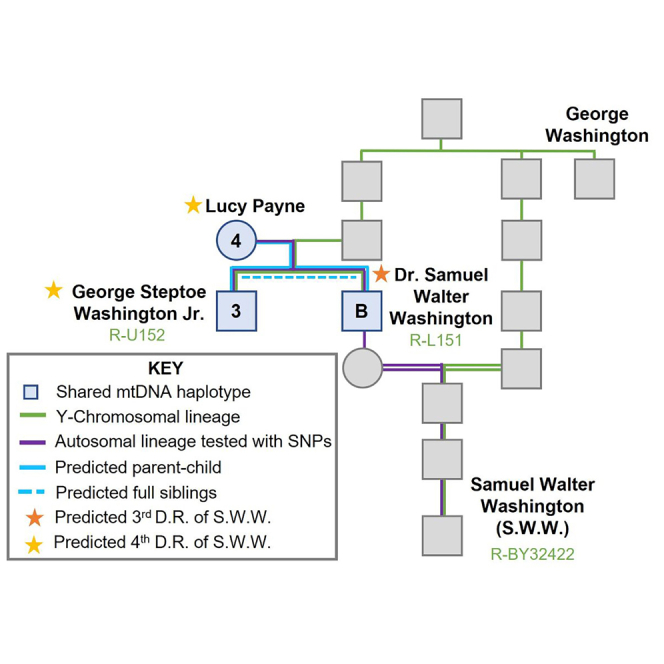

## Introduction

**Figure 1:**
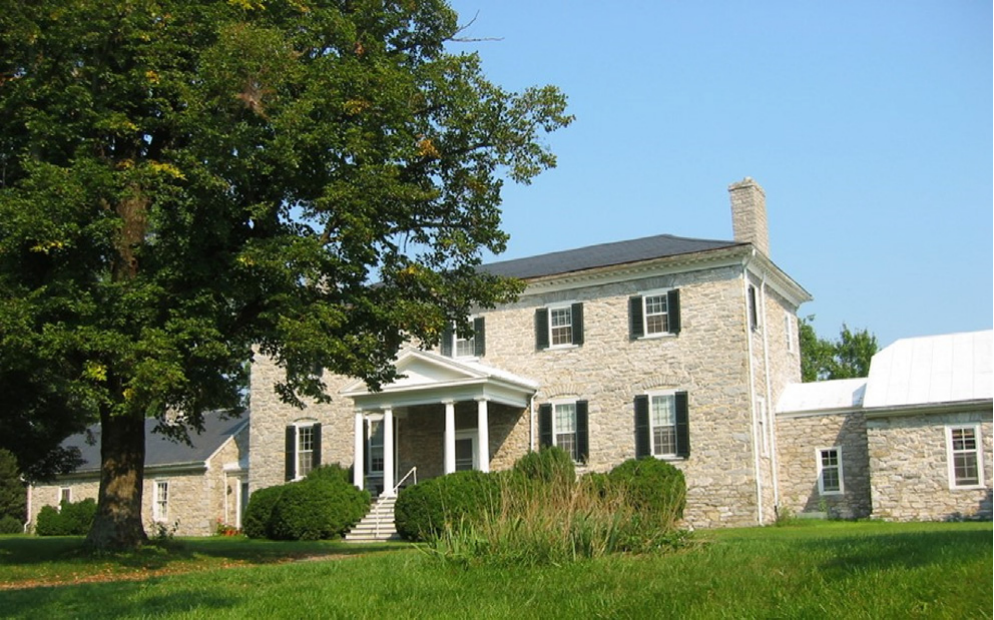
Harewood estate in Charles Town, West Virginia (photo provided by Samuel Walter Washington)

In 1999, an excavation was conducted in Charles Town, West Virginia at Harewood Cemetery with the intent of identifying the unmarked grave of Samuel Washington, the younger brother of President George Washington and former owner of Harewood estate (Figure 1).^1^ The effort was coordinated by James Starrs of George Washington University and volunteer team members from George Washington University, Michigan State University, and the US Armed Forces Institute of Pathology. The excavation was approved by descendants of Samuel Washington for the purpose of identifying unmarked burials. With this permission, a preliminary site survey using ground penetrating radar was conducted to locate points of interest.^2^ Additionally, an archaeological excavation permit was obtained from the West Virginia Division of Culture and History prior to the initial phase of the excavation in May of 1999.

**Figure 2:**
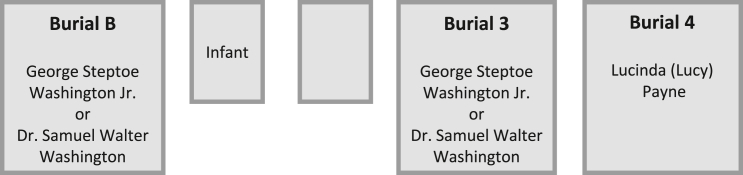
Depiction of unmarked graves and putative identities based on historical records George Steptoe Washington Jr. and Dr. Samuel Walter Washington could be associated with either burial B or 3.

Overall, five unmarked graves were discovered and skeletal remains were collected and cataloged for testing. Primarily, small bones were recovered from the burials, suggesting that these remains may coincide with those disinterred in 1882 for transfer to marked graves at Zion Episcopal Church in Charles Town.^1^ Figure 2 depicts the putative identities of the unmarked remains based on excavation records: George Steptoe Washington Jr., Dr. Samuel Walter Washington, and Lucinda (Lucy) Payne. George Steptoe Washington Jr. and Dr. Samuel Walter Washington could be associated with either burial B or 3. The nomenclature of the burials was maintained from the excavation records.

The Armed Forces Medical Examiners System’s Armed Forces DNA Identification Laboratory (AFMES-AFDIL) was asked to assist with DNA analysis of the skeletal remains. The laboratory was provided with documentation, prepared by members of the Washington family, listing probable burials at Harewood Cemetery to assist with this effort (Table S1). While preliminary research was performed in the early 2000s, this study details more recent testing of the skeletal remains with updated methodologies. Additional testing was approved by Samuel Walter Washington (S.W.W.), who was also involved with the initial excavation, and is a known descendant of Samuel Washington. This study aimed to confirm the identities of the individuals associated with the remains recovered from three burials (3, 4, B; Figure 2) at Harewood Cemetery using historical records and DNA analysis.

Hybridization capture, widely utilized in the field of ancient DNA and more recently forensics, was implemented due to the degraded nature of the remains and the presence of bacterial contamination. This method, combined with next generation sequencing (NGS), has proven to be successful with chemically treated samples that contain <0.01% of human mitochondrial DNA (mtDNA)^3^^,^^4^^,^^5^^,^^6^ and limited quantities of nuclear DNA.^7^^,^^8^ Due to these factors, as well as issues associated with short fragments and cross-linking, these samples were not amenable to traditional short tandem repeat (STR) procedures typically employed in forensics. As a result, two hybridization capture bait panels, one enriching for the mitochondrial genome (mitogenome) and another targeting approximately 95,000 nuclear single nucleotide polymorphisms (SNPs), were utilized to determine the mitochondrial and Y-haplogroups for these samples and to assess kinship between them.

The 95,000 SNP panel (94,752 total SNPs) introduced by Gorden et al. targets 93,559 autosomal SNPs (auSNPs), 448 X-chromosomal SNPs, and 745 Y-chromosomal SNPs (Y-SNPs).^7^ Targeted and non-targeted Y-SNPs were utilized for the Y-haplogroup predictions in the Parabon Fx software (Parabon NanoLabs, Inc., Reston, VA, USA) based on all haplogroup defining SNPs in the ISOGG Y-tree. The Fx analysis software utilizes a probabilistic genotype likelihood approach to analyze targeted auSNPs at a 1X coverage threshold, beneficial for compromised remains similar to those included in this study. Global ancestry (i.e., European, African, Central/South Asian, East Asian, and Latino) is predicted based on auSNP allele frequencies derived from the 1000 Genomes Project, which is then used to select the appropriate allele frequency file for kinship comparisons. Pairwise kinship predictions for each degree of relatedness (i.e., self, parent-child, full siblings, and second through fifth degree) versus unrelated are reported with a likelihood ratio (LR) and posterior probability to determine the strength of each prediction. In the study conducted by Gorden et al., this approach was shown to be reliable and robust when a sufficient number of SNPs were recovered (∼25,000 auSNPs after linkage disequilibrium [LD] pruning).^7^

Non-recombinant Y-chromosomal testing was also performed for haplogroup confirmation and searching of the FamilyTreeDNA database (FTDNA; Houston, TX, USA), which uses the Y Chromosome Consortium (YCC) nomenclature.^9^ auSNP, Y-STR, and Y-SNP data from the bone samples were also compared to data from S.W.W., to evaluate whether the remains belong to the Washington lineage.

**Figure 3:**
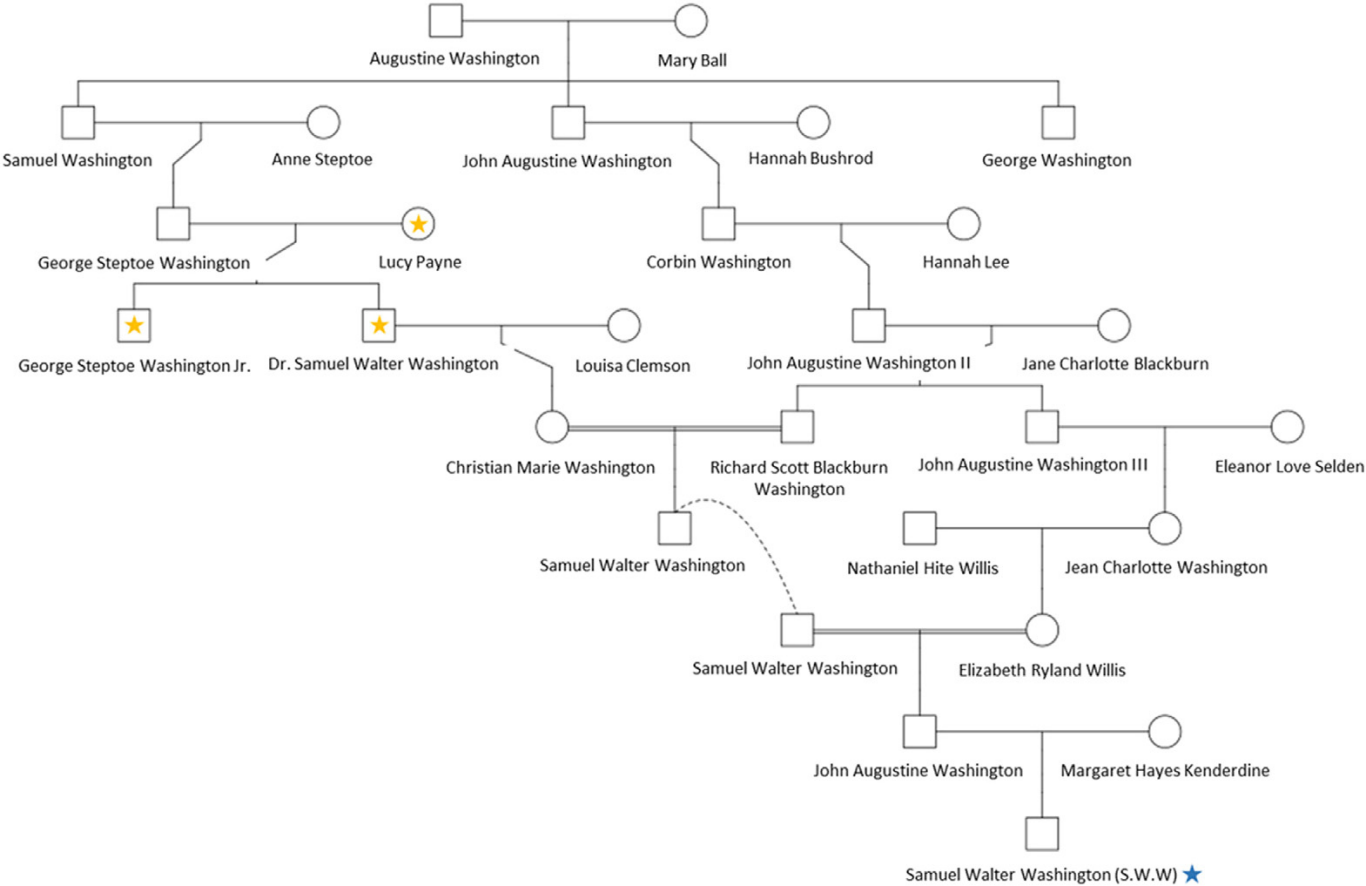
Samuel Walter Washington’s (blue star) family tree limited to ancestors relevant to the relationships discussed in this study The yellow stars represent the individuals believed to be associated with the three burials. The double line is indicative of cross-cousin marriage and the dotted line connects the same individual represented twice within the tree.

The family tree depicted in Figure 3 shows S.W.W.’s relationships to the three buried individuals through multiple lineages as well as President George Washington, based on genealogical records. Lucy Payne is S.W.W.’s great-great-great-grandmother (5^th^ degree relative), George Steptoe Washington Jr. is his great-great-great-uncle (5^th^ degree relative), and Dr. Samuel Walter Washington is his great-great-grandfather (4^th^ degree relative).^10^ S.W.W. is also related to George Steptoe Washington Jr. and Dr. Samuel Walter Washington more distantly, as the result of pedigree collapse caused by cross-cousin marriage, which was confirmed by tracing these lineages through Richard Scott Blackburn Washington and through Elizabeth Ryland Willis. Theoretically, these individuals will exhibit an increased amount of shared DNA that can impact the predicted degrees of relatedness, potentially causing them to appear more closely related than expected.

The Y-STR and Y-SNP data generated from President George Washington’s patriline were also used to infer his Y-chromosomal haplotype, as he had no children of his own. Inference of Washington’s Y-chromosomal haplotype can assist genealogical research in distinguishing genetically versus non-genetically related Washington clusters such as those within the FTDNA database.

## Results

A total of 24 DNA extracts were generated from the skeletal elements recovered from the three burials. At least two skeletal elements were extracted per burial and some were extracted multiple times (STAR Methods, Table 1). At least one standard AFDIL Dabney extract was generated for each burial; however, most extractions adhered to the modified AFDIL Dabney procedure. Individual DNA extracts were not processed through all three testing modalities discussed in the following; however, at least one DNA extract from each burial was tested, resulting in a complete dataset overall (Table S2).

**Table 1:** Skeletal information for the three burials tested

Burial	Sample	Skeletal Element
3	3	Hand Phalange
3	10	Tooth
4	3	Thoracic Vertebra
4	6	Vertebra
4	8	Tooth
B	7	Hamate
B	8	Hand Phalange
B	28	Foot Bone

### DNA quantification

Five DNA extracts (one from burial 3, one from burial 4, and three from burial B) were quantified with Quantifiler Trio. All five DNA extracts produced amplifiable DNA with the 80 bp autosomal target, but only extract B-7A1 resulted in a quantification value for the 214 bp autosomal target (Table 2). Extract 4-6Ac was the only sample that failed to produce amplifiable DNA for the 75 bp Y-DNA target.

**Table 2:** Quantifiler Trio results for DNA extracts that underwent STR typing

DNA Extract	80 bp auDNA (ng/μL)	214 bp auDNA (ng/μL)	75 bp Y-DNA (ng/μL)
3-3Ac	0.00012	UND	0.00014
4-6Ac	0.00024	UND	UND
B-7A1	0.08359	0.00885	0.10483
B-7Ac	0.00376	UND	0.00304
B-28Ac	0.00259	UND	0.00209

UND-undetermined.

### Next generation sequencing

#### Mitogenome capture

Full mitogenome coverage was obtained for extracts 3-3A1, 4-3A1, B-7Ac, and B-8A1, with average coverage ranging from 111 to 747X (Table S3). The mtDNA haplotypes were consistent with one another and belong to mtDNA haplogroup J1c1b1a1, according to EMPOP (Table 3). Extracts B-7Ac and B-8A1 resulted in consistent haplotypes; therefore, only extract B-8A1 was included in Table 3 due to a higher average coverage and less mixed positions due to cytosine deamination (Table S3). Extract 4-3A1 had one mixed position at np 10947 attributable to cytosine deamination that was not observed in the DNA extracts from the other burials. Cytosine deamination was expected in samples of this age and quality. However, this is not considered a difference in the mitogenome profile, since heteroplasmy is generally ignored for haplotype comparisons.

**Table 3:** Mitochondrial DNA haplotypes and haplogroups for DNA extracts 3-3A1, 4-3A1, and B-8A1 that underwent hybridization capture

Burial		
3	4	B
Haplogroup		
J1c1b1a1	J1c1b1a1	J1c1b1a1
73G	73G	73G
185A	185A	185A
228A	228A	228A
263G	263G	263G
295T	295T	295T
462T	462T	462T
315.1C	315.1C	315.1C
482C	482C	482C
489C	489C	489C
750G	750G	750G
1438G	1438G	1438G
2706G	2706G	2706G
3010A	3010A	3010A
3394C	3394C	3394C
4216C	4216C	4216C
4769G	4769G	4769G
5773A	5773A	5773A
7028T	7028T	7028T
7184G	7184G	7184G
8860G	8860G	8860G
10398G	10398G	10398G
10463C	10463C	10463C
	*10947Y*	
11251G	11251G	11251G
11719A	11719A	11719A
12612G	12612G	12612G
13707A	13707A	13707A
13708A	13708A	13708A
14766T	14766T	14766T
14798C	14798C	14798C
15326G	15326G	15326G
15452A	15452A	15452A
15617A	15617A	15617A
16069T	16069T	16069T
16126C	16126C	16126C

Heteroplasmic positions are italicized.

Negative controls (NC) and reagent blanks (RB) processed alongside samples were free of detectable contamination as evidenced by average coverage values <2X (Table S3).

#### 95K SNP capture and kinship prediction with Parabon Fx

Standard AFDIL Dabney extracts (3-3A1, 4-3A1, and B-8A1) resulted in low 95K SNP recovery across the three burials, with the majority of SNPs yielding below 5X coverage (Table S4). Additional processing with modified AFDIL Dabney extracts (3-3A3, 3-10A1, 4-8A1, B-7Ac, and B-28A1) resulted in improved SNP recovery, especially at higher coverage thresholds (Table S4). The S.W.W. reference sample resulted in >94% of the SNPs recovered at the 5X threshold and >88% recovered at 10X. NCs and RBs processed alongside samples resulted in a maximum of 1,270 SNPs covered at 1X (Table S4).

A summary of the 1X SNP recovery results for the best performing DNA extract from each burial and for the S.W.W. sample is presented in Table 4. More than 50% of the total SNPs were recovered for each of the burials and almost complete SNP recovery (99%) was obtained for the S.W.W. reference sample. For the Y-SNP recovery, 745 Y-SNPs were targeted in the 95K SNP capture. Additional, untargeted Y-SNPs were utilized in the Y-haplogroup prediction tool of Parabon Fx; therefore, the targeted Y-SNP recovery as well as the total Y-SNP recovery are both presented in Table 4.

**Table 4:** SNPs covered at 1X after 95K SNP capture, Y-haplogroup and global ancestry predictions for the highest performing DNA extract from each burial and the Samuel Walter Washington (S.W.W.) reference sample

DNA Extract	Total SNPs (% of max 94,752)	Targeted Y-SNPs (% of max 745)	Total Y-SNPs (Including Untargeted)	Y-Haplogroup	Y-Haplogroup alternative nomenclature	Global Ancestry
3-10A1	79,978 (84%)	579 (78%)	2976	R1b1a1b1a1a2b	R-U152	98.96% European
4-8A1	68,103 (80%)	2 (0.27%)	179	None predicted	NA	97.49% European
B-28A1	47,916 (50%)	271 (36%)	768	R1b1a1b1a1a^a^	R-L151	97.30% European
S.W.W.	94,022 (99%)	731 (98%)	11,314	R1b1a1b1a1a2b	R-U152	100% European

Details for all extracts tested with the 95K SNP capture panel are provided in Table S4.

a The finest level of Y-haplogroup resolution due to zero coverage at haplogroup-defining SNPs.

For burial 3, 2976 total Y-SNPs (including 579 out of the 745 targeted Y-SNPs [78%]) were covered at a 1X threshold; for burial B, 768 total Y-SNPs (including 271 of the 745 targeted Y-SNPs [36%]) were covered (Table 4). Burial 4, the presumed female (Lucy Payne), produced two targeted Y-SNPs of the 179 total Y-SNPs, which are likely attributable to erroneous mapping. The S.W.W. sample produced 731 of the 745 targeted Y-SNPs and a total of 11,314 Y-SNPs including those that were untargeted.

The Parabon Fx Y-haplogroup predictions and associated YCC haplogroup nomenclature are included in Table 4. The YCC nomenclature uses the first letter of the major Y-DNA haplogroup to which a Y chromosome belongs and the terminal SNP that defines the branch on which it appears. It is presented along with the Parabon Fx Y-haplogroup predictions to be consistent with the predictions made from Y-STR and Big Y data. The Y-haplogroup predictions were consistent across all DNA extracts for burials 3 and B; however, some predictions were less refined due to lower SNP recovery (Tables S3 and S4).

Y-haplogroup R-U152 was the most refined haplogroup of S.W.W. and burial 3, whereas the most refined haplogroup of burial B was R-L151. A Y-haplogroup was not predicted for burial 4 due to minimal coverage of the Y chromosome despite high SNP recovery on the autosomes and X chromosome. Therefore, burial 4 was likely female and thus represented Lucy Payne, based on the presumed identities of the remains. The global ancestry was predicted to be at least 95% European for all samples with the remaining proportion in burials 3, 4, and B attributable to African ancestry (Tables S3 and S4). As a result, the European frequency file was utilized for all kinship predictions to ensure that the genotype likelihood for the appropriate population was considered.

For each burial, the DNA extract with the highest SNP recovery was selected for kinship prediction (Table S4). Kinship comparisons were performed based on the overlapping auSNPs excluding 23 auSNPs with no allele frequency data in the 1000 Genomes database.

**Figure 4:**
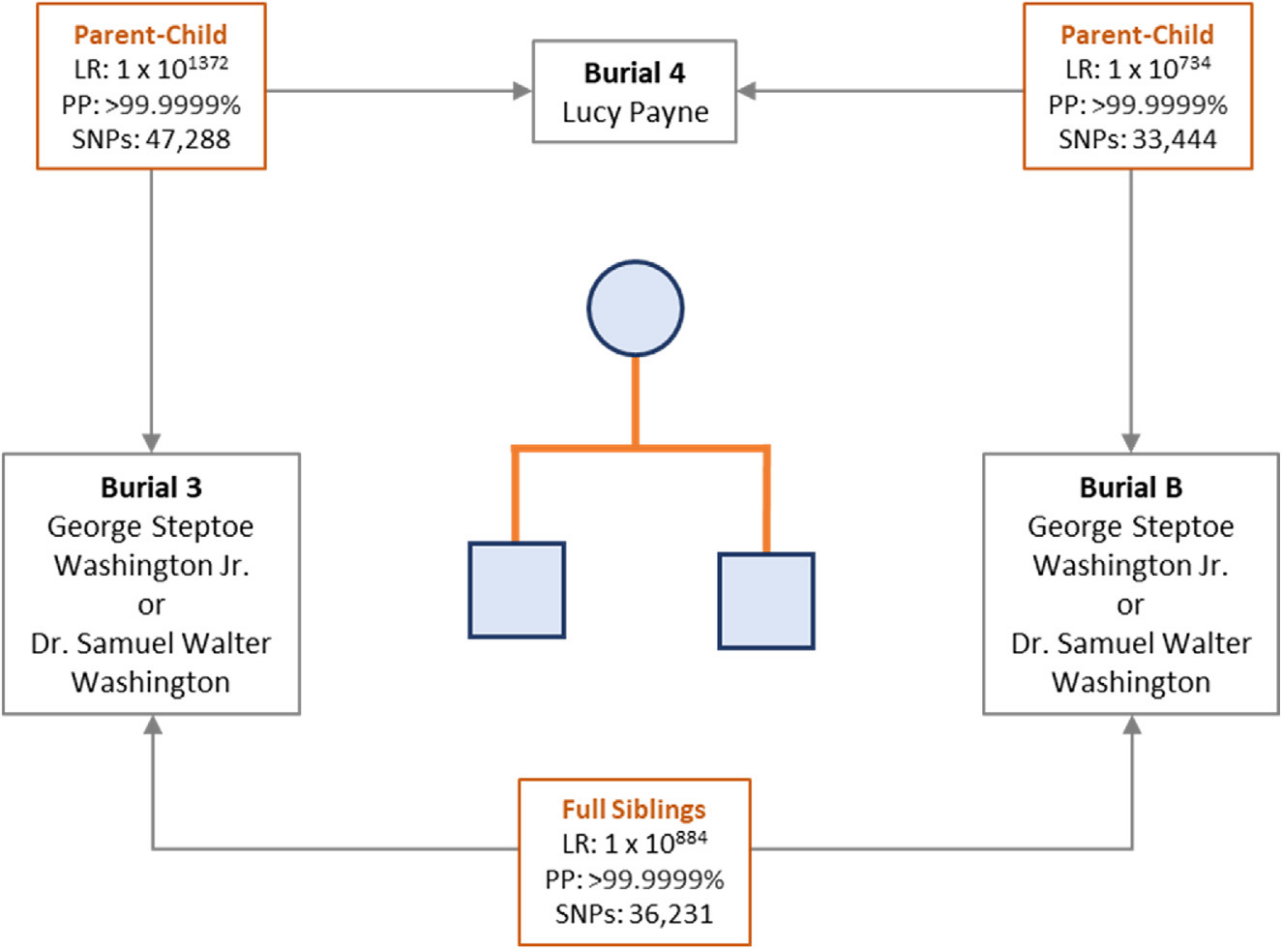
Pairwise kinship predictions and resulting pedigree based on 95K SNP capture data for the three burials LR, likelihood ratio; PP, posterior probability; SNPs, number of overlapping SNPs used for kinship comparison after linkage disequilibrium pruning.

Pairwise kinship predictions were performed among the three burials to assess relatedness. For each burial pair, genotype likelihoods were used to predict relationship likelihoods for each of the following categories: self, parent-child, full siblings, 2^nd^ degree, 3^rd^ degree, 4^th^ degree, 5^th^ degree, and unrelated. The most likely relationship (max category) was reported along with its posterior probability. The LR comparing the most likely relationship versus unrelated was used to demonstrate the strength of the prediction. Among the burials, expected parent-child and full sibling relationships were predicted based on >33,000 overlapping SNPs for each prediction after LD pruning (Figure 4). Burials 3 and B were predicted to be the children of burial 4 and to be full siblings of one another with posterior probabilities exceeding 99.9999% and LRs exceeding 1 x 10^734^. The first degree kinship relationships were confirmed using an alternative forced haploid genome approach with the X-SNPs in the 95K SNP capture panel (Data S1, Figures S1–S6).^11^

**Figure 5:**
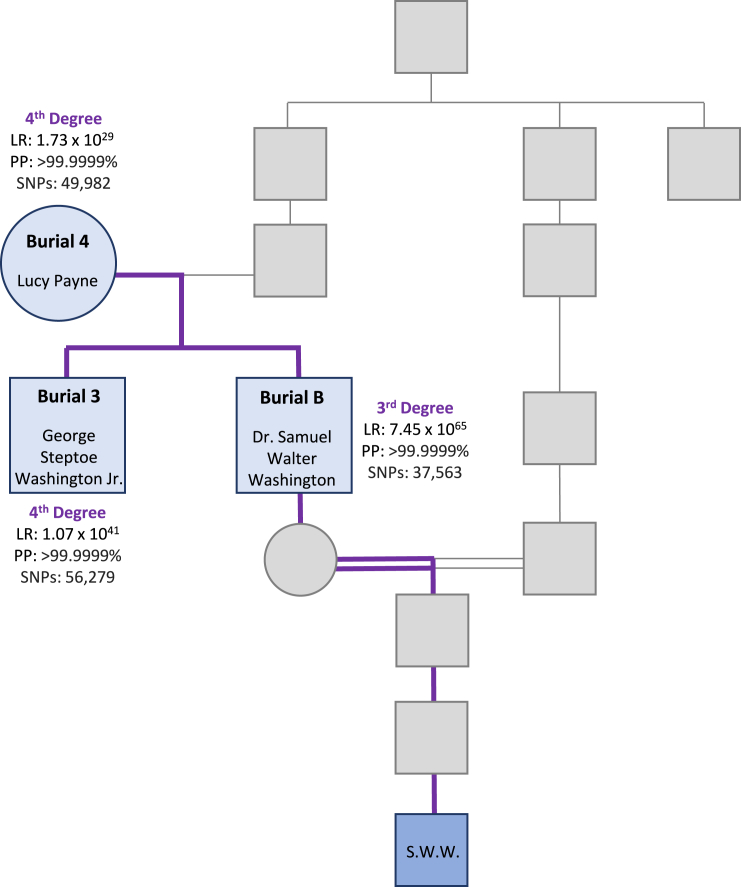
Samuel Walter Washington’s (S.W.W.) pedigree limited to ancestors relevant to the relationships discussed in this study and the results from pairwise kinship predictions between S.W.W. (blue) and the three burials (light blue) The purple line highlights the autosomal lineage evaluated with the 95K SNP capture panel. LR, likelihood ratio; PP, posterior probability; SNPs, number of overlapping SNPs used for kinship comparison after linkage disequilibrium pruning.

Pairwise kinship comparisons were made between S.W.W. and the buried individuals to confirm the Washington family link and to differentiate the identities of the two male burials, since they could not be distinguished with mitochondrial and Y-chromosomal DNA results alone (3 and B; Figure 5). As performed previously, relationships from self to 5^th^ degree relatives were predicted using genotype likelihoods, and the most likely relationship was reported. Burial 4 (the female suspected to be Lucy Payne) was predicted to be a 4^th^ degree relative of S.W.W. based on >49,000 overlapping SNPs, with a posterior probability exceeding 99.9999% when compared to all other relationships (self, parent-child, full siblings, 2^nd^ degree, 3^rd^ degree, 5^th^ degree, and unrelated). The LR of burial 4 being a 4^th^ degree relative of S.W.W. versus unrelated to S.W.W. was 1.73 x 10^29^. Burial 3 was predicted to be a 4^th^ degree relative of S.W.W. based on >56,000 SNPs, with an LR of 1.07 x 10^41^ and a posterior probability exceeding 99.9999%. Burial B was predicted to be a 3^rd^ degree relative with strong statistical support based on >37,000 SNPs, with an LR of 7.45 x 10^65^ and a posterior probability exceeding 99.9999%. The predicted relationships to S.W.W. were one degree closer than expected for all three burials, which is likely attributable to pedigree collapse within the Washington lineage (Figure 3). However, the one-degree difference between burials 3 and B strongly suggests that burial 3 is George Steptoe Washington Jr., S.W.W.’s great-great-great uncle, and burial B is Dr. Samuel Walter Washington, S.W.W.’s closer relative and great-great grandfather.

Given that the 95K kinship comparisons predicted relationships one degree closer than the presumed degree of relatedness, simulation data were generated using the ibdsim2 application to analyze the amount of DNA shared identical-by-descent (IBD) to further support the Fx predictions within the context of the Washington pedigree (Data S2, Figures S7–S10).^12^ The simulated data showed an increase in the number of chromosomal segments shared, which shifted the data associated with the Washington pedigree (Figure 3) toward a closer degree of relatedness (Figures S8 and S9). Thus, the simulations supported the Fx predicted relationships between S.W.W. and his relatives being one degree closer than expected. A shift was not observed in the simulation data for burial 4 (Figure S10), since the two known instances of cross-cousin marriage within the limited Washington pedigree did not impact the amount of shared DNA between Lucy Payne and S.W.W. Additional genealogical research will be required to build out the Payne/Washington pedigree to identify additional instances of pedigree collapse that may have influenced the 95K kinship predictions.

### Y-STR typing

Y-STR typing, using the low copy number Yfiler protocol (LCNY), was performed on at least one extract from each burial for a total of five extracts. Replicate amplifications were carried out for extracts B-7Ac, B-7A1, and B-28Ac due to suspected inhibition based on previous research (Table S2).^13^ Burials 3 and 4 failed to generate reportable profiles (<4 loci), as shown in Table S5. Burial 3 had the lowest quantification result of the four DNA extracts that generated a value for the Y-DNA target (Table 2); therefore, a lower recovery was expected. The Y-STR results and lack of a Y-DNA quantification for burial 4, indicative of the individual being female, further supported the conclusion that the remains belong to Lucy Payne.

Reportable profiles with ≥4 loci for extracts B-7A1, B-7Ac, and B-28Ac (Table S5) were used to generate a composite Y-STR profile for burial B (Table 5). Of the 17 loci included in the assay, 11 alleles were confirmed, one allele was observed below the reporting threshold (>70 relative fluorescence units [RFUs]), and five alleles could not be replicated. The Y-haplogroup was predicted to be R-M269 using all 17 Y-STR loci. Y-haplogroup R-M269 was generally consistent with the Y-SNP haplogroup predictions, although less refined due to a lower number of targeted loci.

**Table 5:** The low copy number Yfiler composite profile for burial B consisting of amplification data from samples B-7 and B-28

Locus	Burial B
DYS456	15^a^
DYS389I	13^a^
DYS390	22
DYS389II	29
DYS458	18^a^
DYS19	14^a^
DYS385	11, 14^a^
DYS393	13^a^
DYS391	10^a^
DYS439	13
DYS635	23^a^
DYS392	13
Y GATA H4	12
DYS437	15^a^
DYS438	12 (BT)
DYS448	19^a^

a Alleles replicated across amplification events and allele 12 at DYS438 was observed below threshold (BT).

The composite profile was compared against the FTDNA database of >600,000 profiles, and one exact match was returned, with an individual having the surname Washington. The DYS635 locus was only included in FTDNA’s Y-111 panel; therefore, an additional search was conducted without this locus for individuals with data from one of the smaller testing kits. This search resulted in additional matches with the Washington surname, with one, two, and three genetic differences.

NCs processed alongside samples were free of detectable contamination with zero reported loci (Table S5). The associated positive controls (PC) generated full profiles consistent with the expected haplotype.

### FamilyTreeDNA Big Y test

The Y-SNP and Y-STR data generated from the S.W.W. reference sample Big Y test were directly compared to Y-DNA data from burial B and the FTDNA database. The Y-SNP haplogroup of S.W.W. was predicted to be R-BY32422, which is more derived than the haplogroups predicted from the burials. This refined haplogroup prediction was possible because of the high number of SNPs (>500,000) in the Big Y test that offers complete Y-haplogroup resolution.

**Figure 6:**
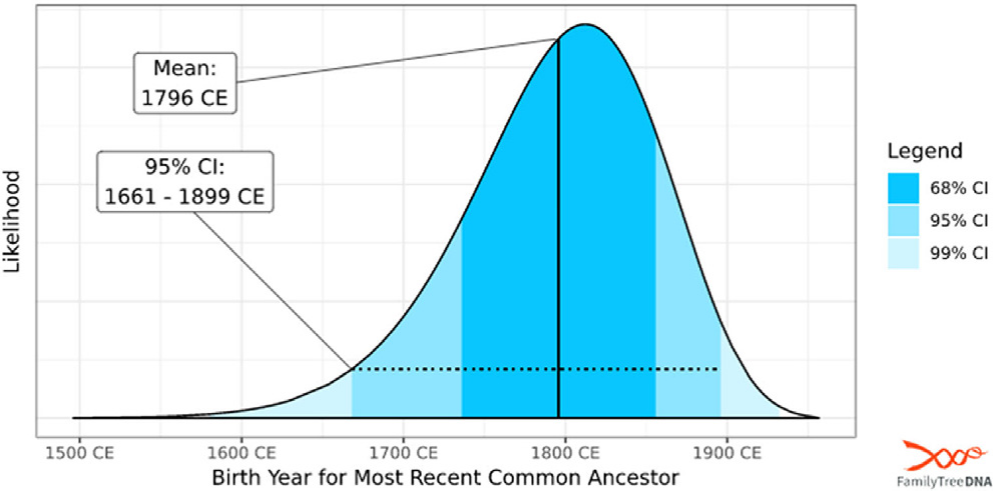
Most recent common ancestor model for Samuel Walter Washington’s closest FamilyTreeDNA match containing two genetic differences across 111 overlapping Y-STRs (Y-111)

The closest matches to S.W.W. in the FTDNA Big Y database (>87,000 profiles), which does not include the two male burials, were about 3,700 years removed; therefore, comparisons were also made using the 111 Y-STR markers tested. The closest match, out of >600,000 profiles, was an individual from the Washington family with a genetic difference of two. Of note, this individual was the exact match to burial B (Dr. Samuel Walter Washington) at all 17 Y-STRs typed in the Yfiler kit. The most recent common ancestor (MRCA) between S.W.W. and the match in FTDNA was likely born between 1661 and 1899 (Figure 6). Additional comparisons using the Big Y profile for S.W.W. were made with 67 and 37 overlapping Y-STRs to account for the smaller panels offered by FTDNA. Three additional matches (two Washingtons and one other) with three, four, and six genetic differences were obtained with 67 overlapping Y-STRs. S.W.W. matched with another Washington descendant with four genetic differences across 37 overlapping Y-STRs.

**Figure 7:**
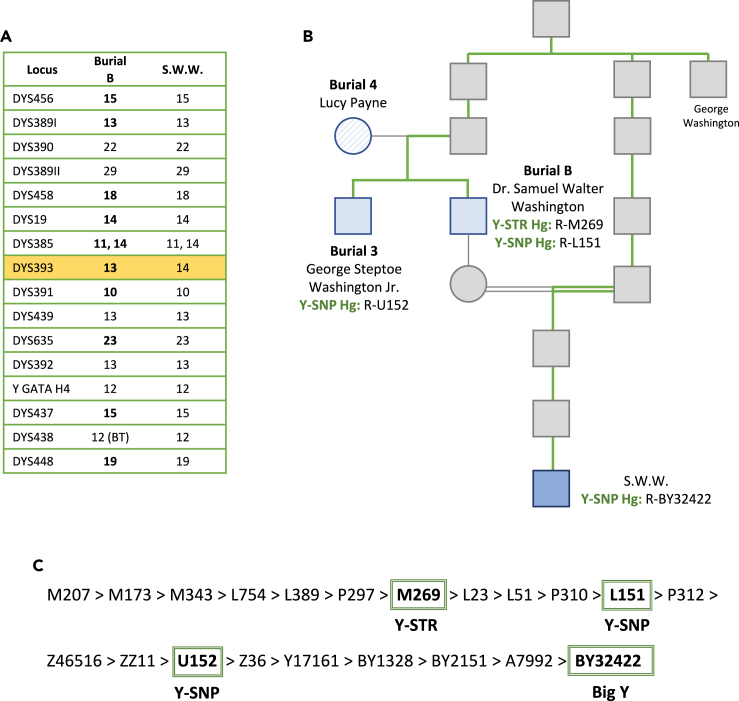
Y-DNA results for the male burials and Samuel Walter Washington (S.W.W.) (A) Comparison of burial B and S.W.W. at the 17 Yfiler loci. The one step difference at DYS393 is highlighted in gold. Alleles in bold font were replicated across amplification events and allele 12 at DYS438 was observed below threshold (BT). (B) Samuel Walter Washington’s (S.W.W.) pedigree limited to ancestors relevant to the relationships discussed in this study. Y-haplogroup predictions generated from Y-SNP and Y-STR data are shown for S.W.W. (blue) and the two male burials (solid light blue). The green line highlights the paternal lineage connecting S.W.W. to the male burials and to President George Washington. (C) The predicted Y-haplogroups for all samples are surrounded by a green box along S.W.W.’s detailed Y-haplogroup pathway. The panel used to generate each Y-haplogroup is listed underneath the prediction.

The Y-STR profiles for S.W.W. and burial B were consistent across every locus but one; therefore, S.W.W. could not be excluded as a possible relative, based on forensic interpretation criteria. At DYS393 S.W.W. had 14 repeats, while the bone sample from burial B had 13 repeats (Figure 7A). S.W.W. and burial B are separated by 10 meioses (Figure 7B), and the observed mutation rate at DYS393 is 0.08%.^14^ The R-M269 Y-haplogroup predicted from the 17-locus Y-STR profile generated for burial B was 14 nodes removed from R-BY32422, which was predicted for S.W.W. based on both Y-STR and Y-SNP markers included in the Big Y assay (Figure 7C). R-BY32422 was also six nodes removed from R-U152 for burial 3 and 10 nodes removed from R-L151 for burial B, both predicted from the 95K SNP capture data (Figure 7C). Despite differences in the Y-STR and Y-SNP haplogroup predictions for the burials, all samples were consistent with the R-BY32422 prediction for S.W.W based on Y-SNP data. Fewer Y-chromosomal markers were targeted and/or covered in DNA testing for the burials; therefore, these Y-haplogroup predictions were less refined as shown in Figure 7C.

## Discussion

This case study highlights the benefits of a multi-marker approach for extended kinship prediction and DNA-assisted identification of historical remains when a reference sample from a living descendant is available. The various testing modalities utilized in this study examined mitochondrial, autosomal, and Y-chromosomal DNA, and supported the putative identities inferred from excavation documents.

Consistent mitogenome haplotypes confirmed that the three individuals were maternally related, while kinship analysis from auSNPs predicted parent-child (burials 3 and 4, burials B and 4) and full sibling (burials 3 and B) relationships with strong statistical support. This information supported the hypothesis that the three individuals associated with the burials are Lucy Payne and her two sons, George Steptoe Washington Jr. and Dr. Samuel Walter Washington. However, mitogenome and 95K SNP capture data from the burials alone could not provide the final piece of the puzzle: the burials with which George Steptoe Washington Jr. and Dr. Samuel Walter Washington were associated with.

The S.W.W. reference sample was integral to the association of burial 3 with George Steptoe Washington Jr. and burial B with Dr. Samuel Walter Washington based on an expected one-degree difference in relatedness. The two brothers could not have been individualized without a direct reference, as they share the same mitochondrial and Y-chromosomal DNA. Kinship comparisons with 95K SNP capture data resulted in relationships one degree closer than anticipated. However, this discrepancy can be attributed to pedigree collapse within the Washington lineage making S.W.W. appear more closely related to the suspected individuals. Two known instances of collapse (i.e., cross-cousin marriage) contributed to an increased amount of DNA sharing between S.W.W. and the male burials in the simulated datasets, supporting this theory. The increased amount of DNA sharing resulted in a shift toward the closer degree of relatedness for each scenario, consistent with the Parabon Fx kinship predictions. The observed ranges of DNA sharing overlap in these more distant relationships. Yet it is also possible that additional, older cross-cousin marriages may exist outside of the Washington pedigree utilized for simulations, since a complete shift was not observed.

Non-recombining Y-chromosomal DNA haplotypes were also integral in confirming that the remains belonged to the Washington lineage. Y-SNP data supported burial 4 being Lucy Payne due to minimal coverage of the Y chromosome in her genomic profile, despite high overall SNP recovery. Extract 4-8A1 had a higher SNP recovery versus extract B-28A1; therefore, one could expect a similar or greater Y-haplogroup score if the individual was male. Additional support was provided by the lack of a Y quantification value and Y-STR typing results. Y-STRs and Y-SNPs obtained from the male burials and FTDNA testers, including S.W.W., provided surname confirmation for the Washington lineage.

Ultimately, the confirmation of the presumed identities for the three burials will allow the remains to be properly buried in a marked grave, as desired by the descendants of Samuel Washington at the outset of the excavation. The presumed identifications based on excavation records and historical context were supported across multiple testing modalities, allowing for a higher degree of confidence in the results. This was made possible in part due to the cross-cousin marriage and two paternal lineages (i.e., Samuel Washington and John Augustine Washington) within the Washington pedigree connecting S.W.W. and the three burials.

Additionally, this study is the first to infer the Y-chromosomal DNA profile of President George Washington’s patriline, via his paternal grandnephews and S.W.W. The inference assumes the absence of an extra-pair paternity (EPP) event impacting the paternity of George Washington; however, the rate of EPP was low in middle to high socioeconomic classes (∼1.0%), which would have included the Washington family.^15^ The Y-haplogroups predicted from the male burials’ Y-SNPs were R-L151 and R-U152, as the most derived haplogroup-defining SNPs with coverage along the Y chromosome. S.W.W.’s more refined Big Y data are associated with R-BY32422, which is supported by both the R-L151 and R-U152 haplogroup-defining SNPs being observed in the historical remains of S.W.W.’s Washington ancestors. This information may be beneficial in resolving multiple Washington clusters when pursuing genealogical research into the founding fathers, since President George Washington did not have children. The Washington surname project within FTDNA consists of 188 members with 40 predicted Y-haplogroups as of June 2023. Therefore, the Y-haplotype of George Washington’s paternal nephews and S.W.W. can be compared to users within the project to determine who is paternally related to George Washington himself.

### Limitations of the study

This study was limited by the Washington family tree utilized to generate simulation data. Building out the Payne/Washington pedigree may identify additional instances of pedigree collapse that could further support the 95K kinship predictions between S.W.W. and the buried individuals.

## STAR★Methods

### Key resources table

**Table:** 

REAGENT or RESOURCE	SOURCE	IDENTIFIER
**Chemicals, peptides, and recombinant proteins**		

Ethanol 200 Proof	Decon Labs, Inc.	Cat#2716
Proteinase K	Promega	Cat#MC5005
AMPure XP	Beckman Coulter	Cat#A63882
2800M Control DNA	Promega	Cat#DD7101
PhiX Sequencing Control v3	Illumina	Cat#FC-110-3001

**Critical commercial assays**		

High Pure Viral Nucleic Acid Large Volume Kit	Roche	Cat#5114403001
EZ1 DNA Investigator Kit	QIAGEN	Cat#952034
Qubit dsDNA High Sensitivity Assay	Thermo Fisher Scientific	Cat#Q32851
Qubit dsDNA Broad Range Assay	Thermo Fisher Scientific	Cat#Q32850
Quantifiler Trio DNA Quantification Kit	Thermo Fisher Scientific	Cat#4482910
KAPA HyperPrep Kit	Roche	Cat#07962371001
KAPA Library Amplification Kit with Primer Mix	Roche	Cat#7958986001
KAPA HiFi HotStart Uracil+ReadyMix Kit	Roche	Cat#7959079001
MinElute PCR Purification Kit	QIAGEN	Cat#28004
KAPA HyperPlus Kit PCR-free	Roche	Cat#7962436001
KAPA HiFi HotStart ReadyMix	Roche	Cat#7958935001
Bioanalyzer High Sensitivity DNA Analysis Kit	Agilent Technologies	Cat#5067-4626
Bioanalyzer DNA Analysis 7500 Kit	Agilent Technologies	Cat#5067-1506
myBaits Expert Mito Kit	Arbor Biosciences	303096.v5
myBaits 95K SNP Kit	Arbor Biosciences	300996R.v5
MiSeq Reagent Kit v3 (150 cycles)	Illumina	Cat#MS-102-3001
NextSeq 500/550 Mid Output Kit 2.5 (300 cycles)	Illumina	Cat#20024905
NextSeq 500/550 Mid Output Kit 2.5 (150 cycles)	Illumina	Cat#20024904
MiSeq FGx Reagent Kit (600 cycles)	Verogen	Cat#15066817
AmpFLSTR Yfiler PCR Amplification Kit	Applied Biosystems	Cat#4359513

**Deposited data**		

FamilyTreeDNA consumer database	goranr@genebygene.com	NA
hg38	UCSC Genome Browser	https://ftp//hgdownload.soe.ucsc.edu/goldenPath/hg38/
Mitogenome and 95K SNP BAM Files	BioStudies	Accession number S-BSST1335

**Experimental models: Cell lines**		

K562 DNA High Molecular Weight	Promega	Cat#DD2011

**Oligonucleotides**		

KAPA Unique Dual-Indexed Adapter Kit	Roche	Cat#08861919702
NEXTFLEX Unique Dual Index Barcodes	Bioo Scientific Corporation	Cat#NOVA-514150

**Software and algorithms**		

CLC Genomics Workbench	QIAGEN	Cat#832022
Parabon Fx Forensic Analysis Platform	Parabon NanoLabs	https://parabon-nanolabs.com/dna-analytics/fx/
GeneMapper® ID-X v1.4	Applied Biosystems	Cat#4479707
NevGen Y-DNA Haplogroup Predictor	NevGen	https://www.nevgen.org/
ISOGG Y-DNA Haplogroup Tree	ISOGG	https://isogg.org/tree/
R v4.0.5	R Core Team, 2021^S3^	https://www.R-project.org/
SNPnexus	Oscanoa, 2020^S5^	https://www.snp-nexus.org/v4/

### Resource availability

#### Lead contact

Further information and request for resources should be directed to and will be fulfilled by the lead contact, Courtney Cavagnino (courtney.l.cavagnino.ctr@health.mil).

#### Materials availability

This study did not generate new unique reagents.

#### Data and code availability

• Mitogenome and SNP data have been deposited at EMBL-EBI’s BioStudies database and are publicly available as of the date of publication. Accession numbers are listed in the key resources table.
• This paper does not report original code.
• Any additional information required to reanalyze the data reported in this paper is available from the lead contact upon request.

### Experimental model and study participant details

#### Skeletal samples

Eight skeletal elements from three burials at Harewood Cemetery in Charles Town, West Virginia were included in this study for DNA analysis (Table 1).

#### Reference sample

To confirm that the excavated remains are Washington descendants, S.W.W., the current owner of Harewood Estate and a member of the Washington family, provided a buccal swab for testing. In reference to the putative identities of the three burials, S.W.W. is a great-great-grandson (4^th^ degree) of Dr. Samuel Walter Washington, a great-great-great-nephew (5^th^ degree) of George Steptoe Washington Jr., and a great-great-great-grandson (5^th^ degree) of Lucy Payne (Figure 3). Also S.W.W. is a “double Washington” via the Samuel Washington and John Augustine Washington lineages (Figure 3). The separate familial branches merged with the marriage of Christian Maria Washington, a direct descendant of Samuel, and Richard Scott Blackburn Washington, a direct descendant of John Augustine. S.W.W. is therefore a great-great-great-great-grandson (6^th^ degree) of Samuel and John, but only a paternal descendant of John Augustine Washington.

#### Institutional review board statement

The study was conducted according to the guidelines of the Declaration of Helsinki and approved by the Defense Health Agency Office of Research Protections on November 20, 2020 (Protocol # DHQ-20-2073).

#### Informed consent statement

The living sample donor provided informed consent for their samples to be used in this project.

### Method details

#### Sample preparation and DNA extraction

Sample preparation and DNA extraction were performed at the AFMES-AFDIL in a low copy, pre-PCR laboratory according to ancient^16^ and forensic^17^ DNA standards. Pre-PCR laboratories with positive pressure, personal protective equipment, and the sterilization (i.e., bleach and/or UV-irradiation) of tools and consumables were implemented to reduce the potential for contamination. Bone samples were sanded to remove exogenous DNA and excess spongy bone. Samples that largely consisted of spongy bone were lightly sanded to retain sufficient bone mass for DNA extraction. These samples underwent a bleach sonication wash prior to standard washes with sterile water and 100% ethanol. Bone samples were then ground to a powder in a Waring blender cup (Waring, Torrington, CT). The bone powder and associated RBs were extracted following the Dabney protocol widely utilized in the ancient DNA community to retain ultrashort DNA fragments.^18^ Protocol modifications were made dependent on whether the standard AFDIL Dabney protocol or modified AFDIL Dabney protocol was used (Table S2). The standard AFDIL Dabney protocol utilized 0.2 g of bone powder, 1 mL of Dabney extraction buffer (0.46M EDTA, 0.05% Tween 20), and 25 μL of proteinase K (20 mg/mL). The modified protocol utilized 1 mL of Dabney buffer per 0.1 g of bone powder and 200 μL of proteinase K to improve digestion. All samples were incubated overnight at 56°C in a Roto-Therm (Benchmark Scientific, Sayreville, NJ) set to 8 rpm on the rock setting. In place of binding buffer ‘D’, 10X Buffer PB (QIAGEN, Hilden, Germany) was added to the supernatant after it was isolated from any remaining powder. Samples were purified through a Roche High Pure Viral silica-based column (Roche, Pleasanton, CA, USA) and Buffer PE (QIAGEN) was used to wash the bound DNA. DNA was eluted in 50–55 μL of Tris-EDTA (10 mM Tris, 0.1 mM EDTA, pH 7.5). Replicate extracts from the same sample were prepared and homogenized, prior to downstream processing, for most samples that underwent NGS and STR typing (Table S2).

Teeth were prepared by wiping the exterior with bleach and 100% ethanol moistened gauze to remove exogenous DNA. The teeth were then UV-irradiated in a PCR hood for 20 min. The crown of sample 3–10 was drilled during a previous round of testing; therefore, the remaining root was powdered in a Waring blender cup. Sample 4–8 was previously bisected, leaving half of the crown and root. A dental burr was used to separate the remaining root from the crown and the root was ground to a powder. Samples were extracted following the modified AFDIL Dabney protocol described previously.

The buccal swab provided by S.W.W. was extracted, alongside an RB, on the QIAGEN EZ1 Advanced XL instrument with the EZ1 DNA Investigator Kit (QIAGEN) according to manufacturer’s recommendations for the Trace protocol. The whole swab was utilized in the extraction and the sample was eluted in 50 μL of sterile water.

#### DNA quantification

The Qubit 2.0 Fluorometer with the Qubit dsDNA HS and BR Assays (all Thermo Fisher Scientific, Waltham, MA, USA) were used to quantify total double-stranded DNA (dsDNA) for all DNA extracts, following the manufacturer’s recommendations.

A subset of DNA extracts that underwent STR typing were also quantified with the Applied Biosystems Quantifiler Trio kit (Thermo Fisher Scientific) on the Applied Biosystems 7500 Real-Time PCR System, according to the manufacturer’s recommendations. The Quantifiler Trio assay consists of a small (80 bp) and large (214 bp) autosomal targets, a 75 bp Y target, and a 130 bp internal positive control.

Two microliters of neat sample extract were used for all quantification assays.

#### Next generation sequencing

##### Library preparation

Sample libraries for the three burials were prepared using the KAPA HyperPrep Kit (Roche Sequencing, Wilmington, MA, USA) with KAPA unique dual-indexed (UDI) adapters (Roche Sequencing), according to the manufacturer’s recommendations, unless noted. No DNA repair was performed prior to library preparation. An NC and PC were initiated at library preparation for each set of samples processed. The PC consisted of 1 ng of enzymatically fragmented K562 cell line DNA (Promega Corporation, Madison, WI, USA).^3^ Qubit values were used to determine total dsDNA input for library preparation, with a maximum allowable input of 1 μg. A reduced total dsDNA input was used for extract B-7Ac due to signs of inhibition observed in previous research.^13^ At adapter ligation the stock concentration (15 μM) of UDI adapter was used for all samples and RBs. NCs and PCs received a 1.5 μM dilution due to a dsDNA input ≤1ng. Following adapter ligation, a 1.3x AMPure XP (Beckman Coulter, Brea, CA, USA) purification was performed for small fragment retention. Library PCR was performed using KAPA HiFi HotStart Uracil+ Ready Mix and 20 μM Illumina Primer mix (Roche Sequencing) with the manufacturer’s recommended cycling parameters for a total of 12 PCR cycles. Library PCR product was purified using a 5X MinElute (QIAGEN) purification and libraries were eluted in 25 μL Tris-EDTA.

The reference sample library was prepared using the KAPA HyperPlus Kit (Roche Sequencing), according to the manufacturer’s recommendations, unless noted. Qubit values were used to determine total dsDNA input into fragmentation, with a maximum allowable input of 1 μg. An NC and PC (Promega 2800M, 45 ng input) were initiated prior to library preparation, which started with a 15-min fragmentation step. BIOO NextFlex single end 12nt indexed adapters (BIOO Scientific, Austin, TX, USA) were incorporated at adapter ligation. The reference sample was amplified with KAPA HiFi HotStart ReadyMix (Roche Sequencing) and 20 μM Illumina Primer Mix, following the same cycling parameters as the HyperPrep samples. The reference sample library was purified using a 5X QIAGEN MinElute, with elution in 25 μL 10 mM Tris-HCl.

Resulting library quality was assessed on the Agilent 2100 Bioanalyzer instrument (Agilent Technologies, Santa Clara, CA, USA) for all samples.

##### Hybridization capture

Hybridization capture was performed on purified libraries with two separate bait panels, using the myBaits v5 kit (Arbor Biosciences, Ann Arbor, MI, USA). Individual sample libraries were not captured using both bait sets (Table S2); however, at least one extract from each of the burials was captured with the mitogenome and 95K bait sets, resulting in complete datasets overall. A predesigned Global Mito bait set was used to target the human mitogenome for all samples except B-7.^19^ Sample B-7 was captured with a custom human mitogenome panel as part of a different sample set.^3^ The 95K panel contains 94,752 nuclear SNPs, including 93,559 auSNPs, 448 X-SNPs, and 745 Y-SNPs, with two baits covering each target.^7^ The reference sample was only captured with the 95K panel for the purpose of kinship comparison. No mitogenome data were generated for the S.W.W. sample, since the reference is not maternally related to the burials.

Hybridization capture and post capture purification were performed according to Thomas et al.^13^ Purified capture libraries were analyzed on the 2100 Bioanalyzer instrument using the DNA 7500 or DNA High Sensitivity assay (both Agilent Technologies).

##### Pooling and sequencing

Mitogenome captured libraries were primarily pooled by volume and the pool was quantified on the Agilent Bioanalyzer 2100 instrument with the DNA 7500 kit. The library pool was diluted to 4 nM based on the molarity detected in the 100–7500 bp region. The 4 nM pool was denatured and further diluted to an 8 pM pool spiked with 2.5% PhiX Control v3 (Illumina, San Diego, CA, USA). Paired end sequencing (75x75 cycles) was performed on a MiSeq FGx (Verogen, San Diego, CA, USA) with a 150 cycle v3 MiSeq reagent kit (Illumina). Sample B-7 was sequenced as part of a larger set with a range in sample qualities; therefore, samples libraries were normalized to 50 nM based on the 170–1000 bp molarity of the Bioanalyzer DNA 7500 kit. Following normalization, samples were pooled by equal volume and diluted to 4 nM based on the Bioanalyzer quantification. The denatured 4 nM pool was further diluted to 1 pM with 2.5% PhiX. Paired end sequencing (150x150 cycles) was performed on a NextSeq 550 (Illumina) using a Mid 300 Output v2.5 Kit (Illumina).

95K captured libraries, generated with standard AFDIL Dabney extracts, were individually normalized to 4 nM and pooled. A 1.2 pM sample pool with 10% PhiX was sequenced on a NextSeq Mid 150 v2.5 kit (Illumina) with 75x75 cycle paired end sequencing. The 95K capture libraries generated with modified AFDIL Dabney extracts were similar in molarity and thus pooled by volume. A 1.0 pM sample pool with 10% PhiX was loaded using the same parameters as the initial 95K sequencing run.

PCs were not sequenced for the capture sets that included libraries from the three burials to prevent crosstalk and were only utilized to assess library preparation and hybridization capture success.^3^

The 95K hybridization capture library for the reference sample was pooled by volume with all associated controls (including the PC) and diluted to 4 nM, as described previously. A 12 pM pool with 5% PhiX was sequenced on a MiSeq FGx reagent cartridge (Illumina) with 300x300 cycle paired end sequencing.

##### Sequence data analysis and kinship comparison

Demultiplexed FASTQ files were imported into the CLC Genomics Workbench v12.0.1 (QIAGEN) with the AFDIL-QIAGEN mtDNA Expert (AQME) plug-in for mitogenome analysis using a custom workflow.^3^^,^^20^ Reads were mapped to the revised Cambridge Reference Sequence (rCRS)^21^^,^^22^ with stringent parameters (0.85 length fraction and 0.95 similarity fraction) to minimize non-specific reads. De-duplication and indel alignment were performed using a custom de-duplication tool with local realignment. Variants were called if the position exceeded a 10X coverage threshold with a variant frequency ≥10% and minimum variant count of 4. NCs and RBs were considered clean if the average coverage was <2X, as determined by the limit of quantitation observed in a set of clean controls processed as part of an internal validation. EMPOP (v4/R13) was utilized to estimate the mtDNA haplogroup for all samples tested.^23^

SNP data generated with the 95K capture panel were imported into the Parabon Fx Forensic Analysis Platform and analyzed according to Gorden et al.^7^ Reads were aligned to the hg38 human reference genome with the following parameters and penalties: assess damage, remove duplicates, gap extension penalty = 1, gap open penalty = 6, mismatch penalty = 4, and unpaired read penalty = 9. SNP profiles were generated with a coverage threshold of 1X since the software uses a probabilistic genotype likelihood approach for analyzing targeted SNPs.^7^ Damage correction was applied to the historical samples during SNP capture profile generation due to the degraded nature of the remains. The probability of cytosine deamination at each base position was factored into the genotype likelihood calculation based on the mismatch rate at the 5′ and 3′ ends of the fragments that exhibited cytosine deamination patterns.^7^ In addition to the 745 Y-SNPs targeted by the 95K SNP panel, Y-haplogroup predictions performed in Parabon Fx are based on all Y-haplogroup defining SNPs from the ISOGG Y tree (accessed April 2, 2020). The YCC nomenclature was also determined for the 95K SNP data to be consistent with the nomenclature used for the Y-STR and Big Y haplogroup predictions.

For auSNPs, ancestry predictions were performed in Fx based on allele frequencies incorporated in the 1000 Genomes Project.^7^ The predicted ancestry for each sample was utilized for the allele frequency file in pairwise kinship analyses amongst the three skeletal samples and reference sample captured with the 95K SNP panel. Pairwise comparisons, utilizing the 93,536 kinship SNPs (excludes 23 auSNPs not represented in 1000 Genomes), were performed to determine the likelihood of each relationship (i.e., self, parent-child, full siblings, and second through fifth degree) between two samples as described in Gorden et al.^7^ These comparisons incorporated damage correction for the burial samples and bioinformatic pruning of overlapping SNPs in LD (R^2^ > 0.2).^7^ The resulting LRs and posterior probabilities for each degree of relatedness versus unrelated were evaluated to assess the strength of each comparison. Kinship predictions were accompanied by strong statistical support if the following criteria were met: LR ≥ 1 x 10^4^ and a posterior probability ≥99.99% (assuming a flat prior).^8^

#### Forced haploid approach

There are a number of alternative approaches available to estimate the degree of relatedness from low coverage DNA data. One category is based on the analysis of allele sharing proportions between pairs of datasets.^11^^,^^24^^,^^25^ The “forced haploid approach” was applied to the SNP data obtained herein.^11^ This method measured the observed allele mismatch proportion between pseudo-haploid genomes of the tested samples. This mismatch proportion was then combined with a simulation approach to obtain expected mismatch distributions for various degrees of relatedness with the same set of SNPs. The mismatch proportion depends not only on the degree of relatedness, but also on the heterozygosity of the included SNPs (i.e., population allele frequency distributions). The implementation of SNP specific population allele frequencies, specified pedigrees, and an error model to account for possible genotype errors in the datasets, allowed the simulation model to produce estimates of expected mismatch distributions from which the degree of relatedness could be inferred.

Inheritance patterns differ between autosomal and X-chromosomal DNA markers; therefore, different allele sharing proportions are expected when analyzing the same relationship with different types of SNP data. Furthermore, for X-chromosomal data mismatch proportions are influenced by the sex of the tested individuals. The expected mismatch proportion distributions for first degree relationships, such as parent/child and full siblings, will overlap for autosomal markers, while these proportions are differentiated for X-chromosomal markers.

The mismatch proportion was estimated as follows: For each pair (burial 3 vs. 4, burial 3 vs. B and burial 4 vs. B), the original SNP read data observations were reduced to SNPs that overlapped between the two samples (e.g., SNPs with no reads in one or both samples were excluded). Next, using a custom R script, the datasets were forced into pseudo-haploid genomes followed by a comparison and calculation of the allele mismatch proportion.^26^ The resulting SNP marker set (i.e., SNPs with allele calls in both samples) was then used as the input for the mismatch proportion simulation method. To obtain the expected mismatch proportion distributions, pseudo-haploid genome datasets for various degrees of relatedness (i.e., same individual, parent/child, full siblings, or unrelated individuals for autosomal SNPs and parent/child, full siblings and unrelated for X-chromosomal SNP data) were simulated using European allele frequencies from the Genomes Project Consortium and SNP-nexus for the specific set of SNPs that were included in each of the sample comparisons.^27^^,^^28^ In addition, a simplistic error model was included to account for the possibility of different genotype errors (e.g., deamination, depurination, PCR error, sequencing error and mapping error) that may occur for low-quality and low-quantity DNA samples. Two different error rates (0 and 0.005) were used in the simulations.

#### Pedigree simulations

Simulation data were generated using the ibdsim2 application of ped suite to compare the amount of DNA shared identical-by-descent (IBD) between the two male burials, and the two male burials versus S.W.W. to further support the Fx predictions within the context of the Washington pedigree (Figure 3).^12^ Chromosomal DNA segments shared IBD are inherited from a common ancestor. The amount of shared DNA and the length of the shared segments can be indicative of population size and distance to the MRCA.^29^ Smaller populations will exhibit a higher degree of IBD sharing and longer chromosomal segments suggest a more closely related MRCA.

Simulation data for each of the scenarios detailed below were generated with the following parameters: 1000 datapoints, chi2 statistical model, and decode (1–22) genetic mapping. Sex specific recombination rates were not considered and a centimorgan (cM) cutoff was not applied to the dataset. A multivariate normal distribution was assumed for each set of 1000 datapoints. An ellipse, with a confidence level of 0.99, was drawn based on the distributions and any datapoints falling outside were considered extremes.

Simulation data were generated for burials 3 and B in the Washington pedigree, which were predicted to be full siblings based on the Parabon Fx kinship predictions with 95K SNP data. Data were compared to pedigrees for full siblings (1^st^ degree) and half siblings (2^nd^ degree), to prove that the 1^st^ degree relationships predicted in Parabon Fx were reliable, despite closer than anticipated predictions for the more distant relationships with S.W.W.

Simulation data were then generated for each burial using the Washington pedigree (with pedigree collapse) and two additional pedigrees, depicting the appropriate expected (without pedigree collapse) and Fx predicted degrees of relatedness, to accurately reflect the possible amounts of DNA sharing. Data generated from the Washington pedigree for burial 3, identified as George Steptoe Washington Jr. based on the Parabon Fx kinship predictions, were compared to pedigrees for a great-great-uncle (4^th^ degree relative) and a great-great-great-uncle (5^th^ degree relative). Burial B, identified as Dr. Samuel Walter Washington, was compared to pedigrees for a great-grandfather (3^rd^ degree relative) and a great-great-grandfather (4^th^ degree relative). Burial 4, identified as Lucy Payne, was compared to pedigrees for a great-great-grandmother (4^th^ degree relative) and a great-great-great-grandmother (5^th^ degree relative).

#### Y-STR typing

STR typing was performed using the AmpFLSTR Yfiler kit (Applied Biosystems), targeting 17 Y-STR loci, and an LCNY protocol developed at AFMES-AFDIL for highly degraded, low copy number remains.^30^ A range of DNA inputs were amplified for samples B-7 and B-28 with suspected inhibition based on previous testing.^13^ An NC and 2800M PC were included with each amplification event.

Samples were prepared for typing on a 3500xL Genetic Analyzer with POP-4 polymer (Applied Biosystems), according to the manufacturer’s instructions. All samples were injected for 7 seconds with default parameters.

Data were analyzed in GeneMapper ID-X v1.4 (Applied Biosystems) and peaks over 70 RFUs were considered reportable. Composite profiles were generated for samples with at least two amplifications containing ≥4 reportable loci. The Y-haplogroup was predicted using NevGen’s Y-DNA haplogroup predictor. The LCNY composite profile was also compared against the FTDNA database of >600,000 profiles for possible surname identification. The genetic difference for each database match was calculated as the total number of differences in STR repeats. For example, a genetic difference of two can be the result of a two-step mutation at a single locus or a one-step mutation at two loci.

#### FamilyTreeDNA Big Y test

The S.W.W. reference sample was also processed through FTDNA’s Big Y and the results were compared to FTDNA’s database.^31^^,^^32^ The Big Y test targets 700 Y-STRs and a region consisting of 590,000 known Y-SNPs. The Y-STR data within the Big Y test was also compared to LCNY data obtained from the burials.

## Supplemental information

Document S1. Figures S1–S10 and Data S1 and S2

Table S1. Probable burials at Harewood Cemetery from oldest to most recent burial, related to Figure 2 Burials highlighted in light blue were moved to Zion Church circa 1882 and burials highlighted in light gray have a tombstone at Harewood Cemetery.

Table S2. Sample processing overview for individual extracts, related to Table 5 Extract numbers with a “c” indicate that two separate extracts were prepared and homogenized prior to downstream processing. Mitogenome,mitochondrial genome; LCNY, low copy number Yfiler protocol.

Table S3. Mitogenome capture metrics and haplotypes for samples, negative controls (NC), and reagent blanks (RB), related to Table 2 Heteroplasmic positions are italicized.

Table S4. 95K SNP capture metrics for the burials, the Samuel Walter Washington (S.W.W.) reference sample, and associated controls, related to Table 3

Table S5. LCNY amplification data for individual sample replicates, negative controls (NC), and positive controls (PC), related to Table 4 BT, below threshold.
