# Supplementary material for: Unearthing who and Y at Harewood Cemetery and inference of George Washington’s Y-chromosomal haplotype
Source: iScience. 2024 Mar 28;27(4):109353. doi: 10.1016/j.isci.2024.109353 (PMC11074960; doi:10.1016/j.isci.2024.109353)
Supplement: Document S1. Figures S1–S10 and Data S1 and S2 [file mmc1.pdf]

iScience, Volume *27*

Supplemental information

Unearthing who and Y at Harewood Cemetery and inference of George Washington's Y-chromosomal haplotype

Courtney Cavagnino, Göran Runfeldt, Michael Sager, Roberta Estes, Andreas Tillmar, Ellen M. Greytak, Jacqueline Tyler Thomas, Elise Anderson, Jennifer Daniels-Higginbotham, Katelyn Kjelland, Kimberly Sturk-Andreaggi, Thomas J. Parsons, Timothy P. McMahon, and Charla Marshall

Supplemental Data S1: Forced haploid approach

Autosomal SNP data

The observed mismatch proportions for burial 3 vs 4, burial 3 vs B and burial 4 vs B, were estimated to 0.244, 0.240 and 0.238 respectively, which are consistent with the expected mismatch proportion for first degree relationships such as parent/child and full siblings (Figures S1-S3). Thus, the results from the forced haploid genome analyses, based on the autosomal SNP markers, confirm a first-degree relationship between burials 3, 4 and B, but the specific type of such relationship could not be inferred.

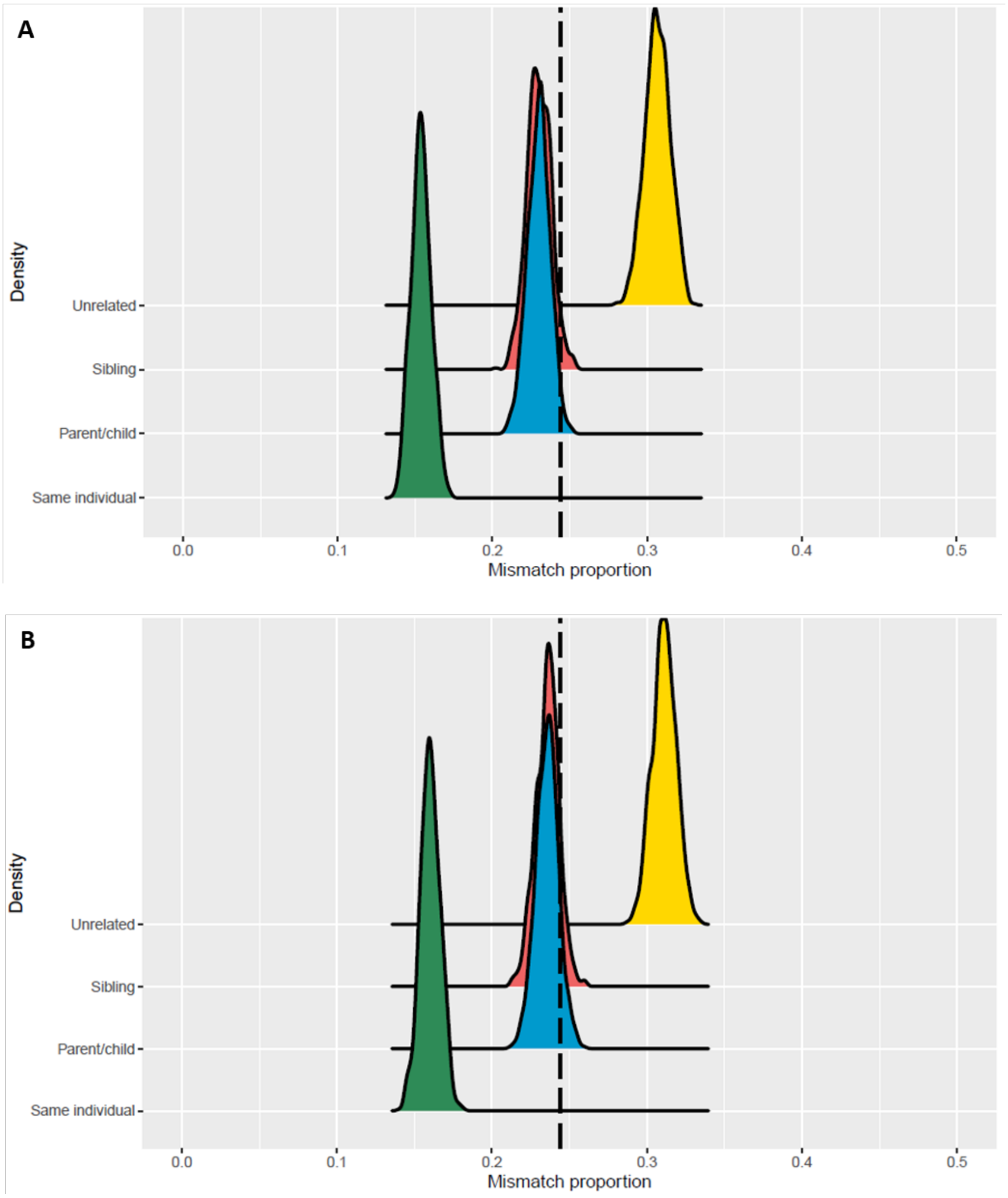

Figure S1. Observed mismatch proportions for burial 3 vs 4 comparisons (black dashed lines) in relation to the expected mismatch proportions (distribution plots) for various degrees of relatedness with (A) error rate= 0 and (B) error rate= 0.005.

These plots are based on the analyses of autosomal SNP data. Green= same individual, blue= parent/child, red= sibling, yellow= unrelated, Related to Figure 4

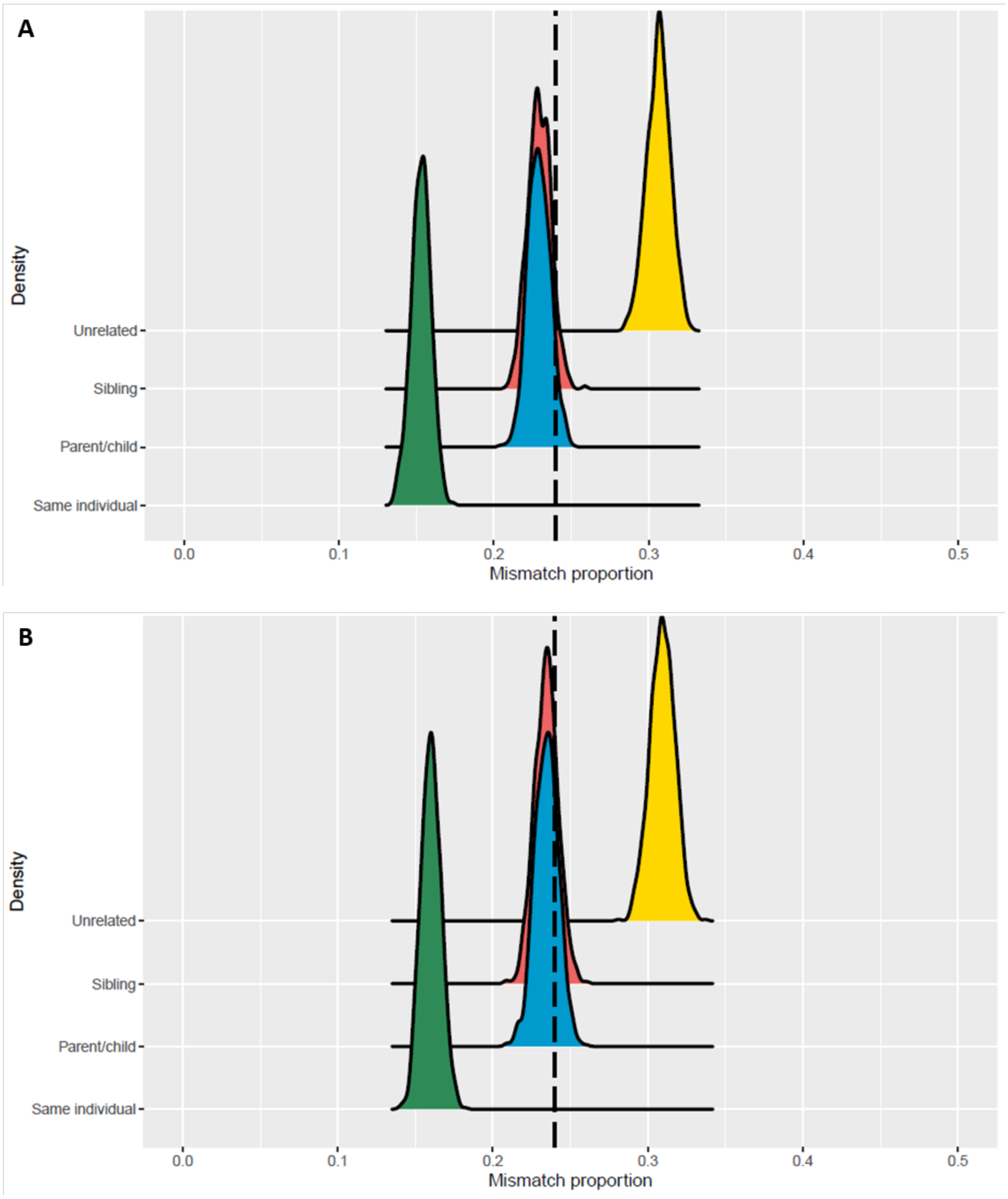

Figure S2. Observed mismatch proportions for burial 3 vs B comparisons (black dashed lines) in relation to the expected mismatch proportions (distribution plots) for various degrees of relatedness with (A) error rate= 0 and (B) error rate= 0.005. These plots are based on the analyses of autosomal SNP data. Green= same individual, blue= parent/child, red= sibling, yellow= unrelated, Related to Figure 4

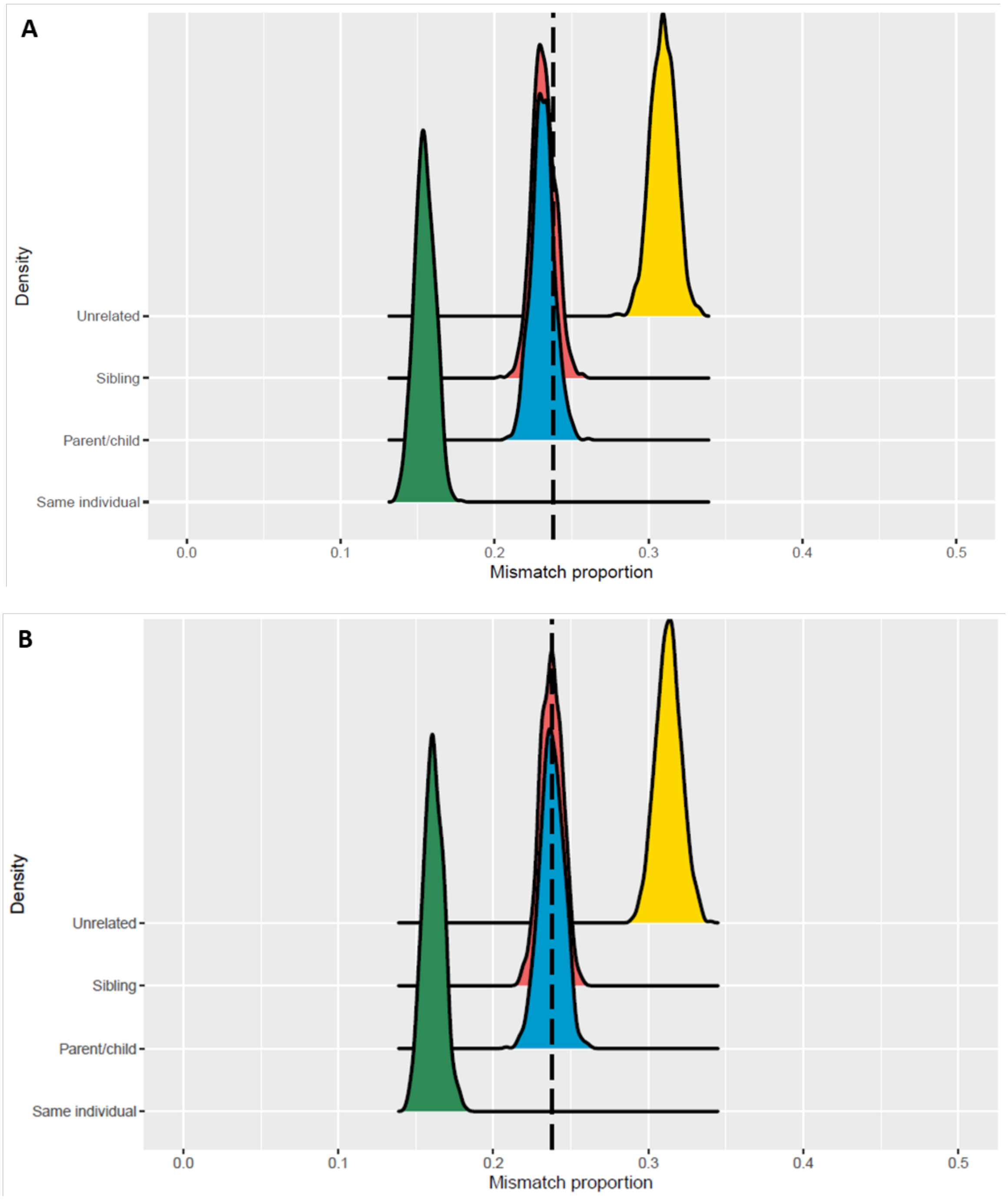

Figure S3. Observed mismatch proportions for burial 4 vs B comparisons (black dashed lines) in relation to the expected mismatch proportions (distribution plots) for various degrees of relatedness with (A) error rate= 0 and (B) error rate= 0.005. These plots are based on the analyses of autosomal SNP data. Green= same individual, blue= parent/child, red= sibling, yellow= unrelated, Related to Figure 4

X-chromosomal SNP data

Since the forced haploid genome analysis based on autosomal SNP markers confirmed a first-degree relationship but could not distinguish between parent/child and full siblings, additional analyses were performed using X-chromosomal SNP data. Different inheritance patterns are expected for different first-degree relationships, which also are affected by the sex of the compared individuals.

The observed mismatch proportion for burial 3 vs 4 was estimated to 0.20. This mismatch proportion is consistent with the expected proportions for a parent/child relationship and differs from the expected proportions for a full sibling relationship ($P<0.05$) (Figures S4-S6).

The observed mismatch proportion for burial 3 vs B was estimated to 0.15. This mismatch proportion is consistent with the expected mismatch proportions for a full sibling relationship and differs from expected proportions for a parent/child relationship ($P<0.05$) (Figure S2).

The observed mismatch proportion for burial 4 vs B was estimated to 0.21. This mismatch proportion is in agreement with the expected mismatch proportions for both a parent/child relationship and a full sibling relationship (Figure S2). For this pair, data from more X-SNPs (161 X-SNPs were included in this comparison) is needed to differentiate expected mismatch proportions for these relationships.

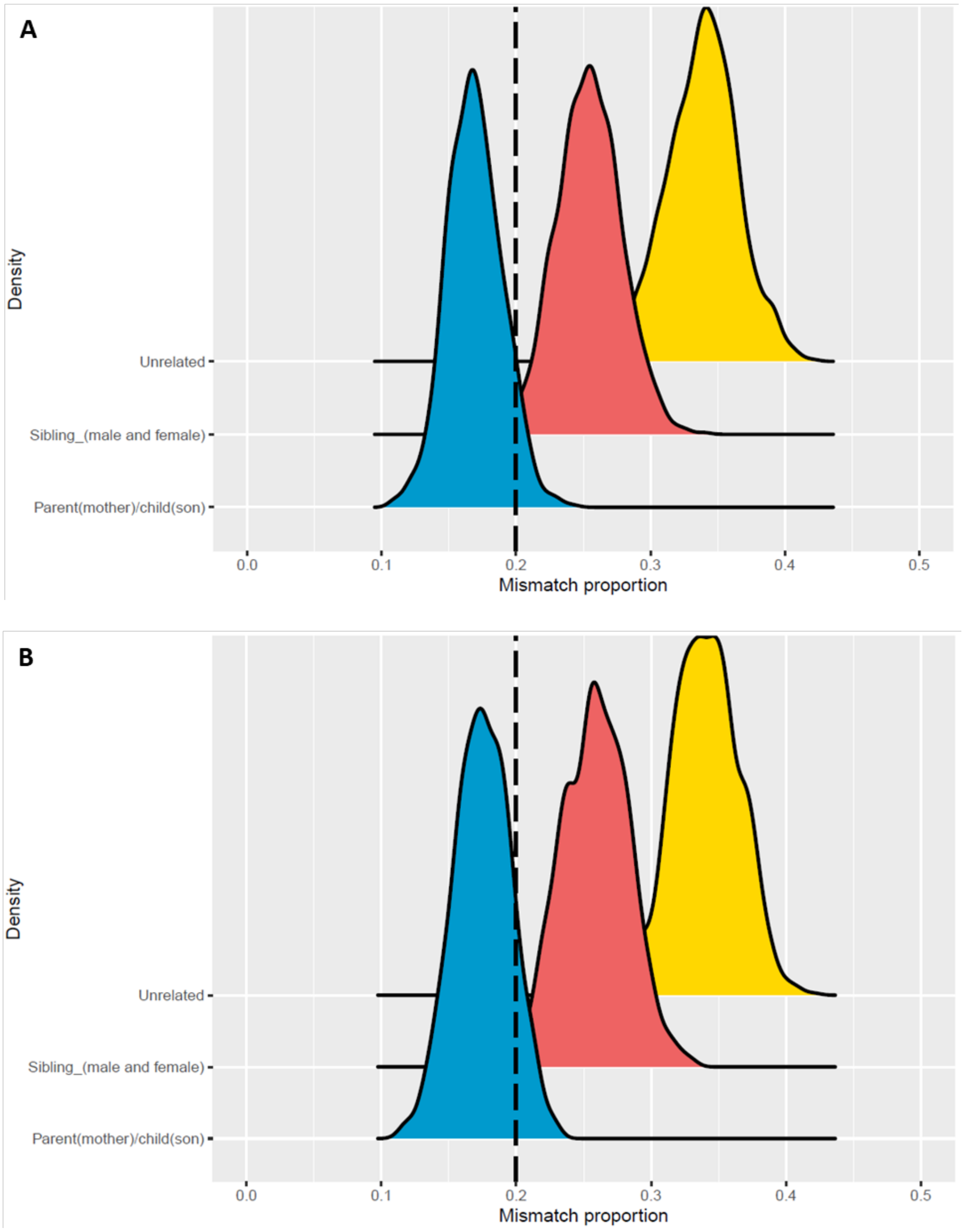

Figure S4. Observed mismatch proportions for burial 3 vs 4 comparisons (black dashed lines) in relation to the expected mismatch proportions (distribution plots) for various degrees of relatedness with (A) error rate= 0 and (B) error rate= 0.005. These plots are based on the analyses of X-chromosomal SNP data. Blue= parent (mother)/ child (son), red= sibling (male and female), yellow= unrelated (male and female), Related to Figure 4

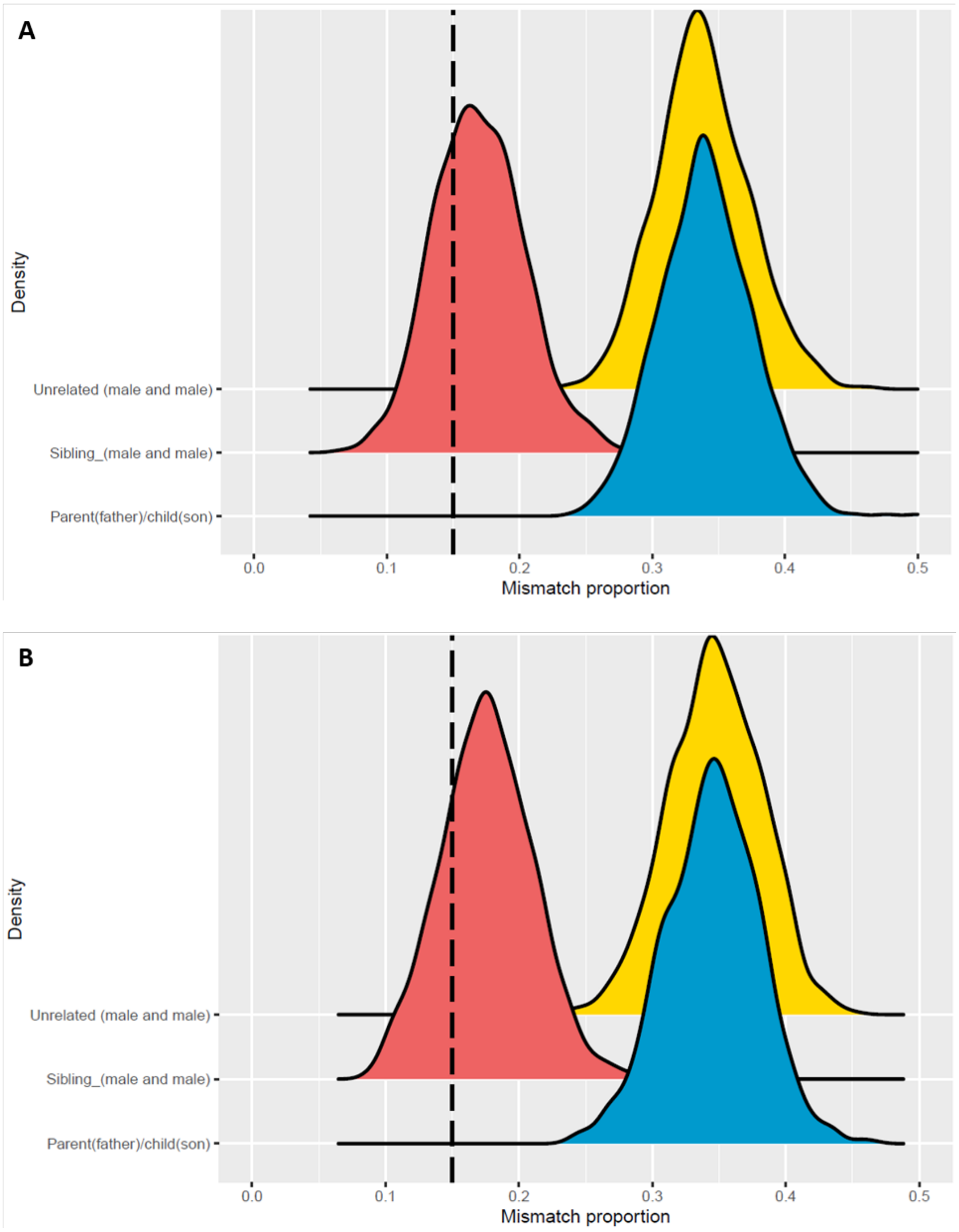

Figure S5. Observed mismatch proportions for burial 3 vs B comparisons (black dashed lines) in relation to the expected mismatch proportions (distribution plots) for various degrees of relatedness with (A) error rate= 0 and (B) error rate= 0.005. These plots are based on the analyses of X-chromosomal SNP data. Blue= parent (father)/ child (son), red= sibling (male and male), yellow= unrelated (male and male), Related to Figure 4

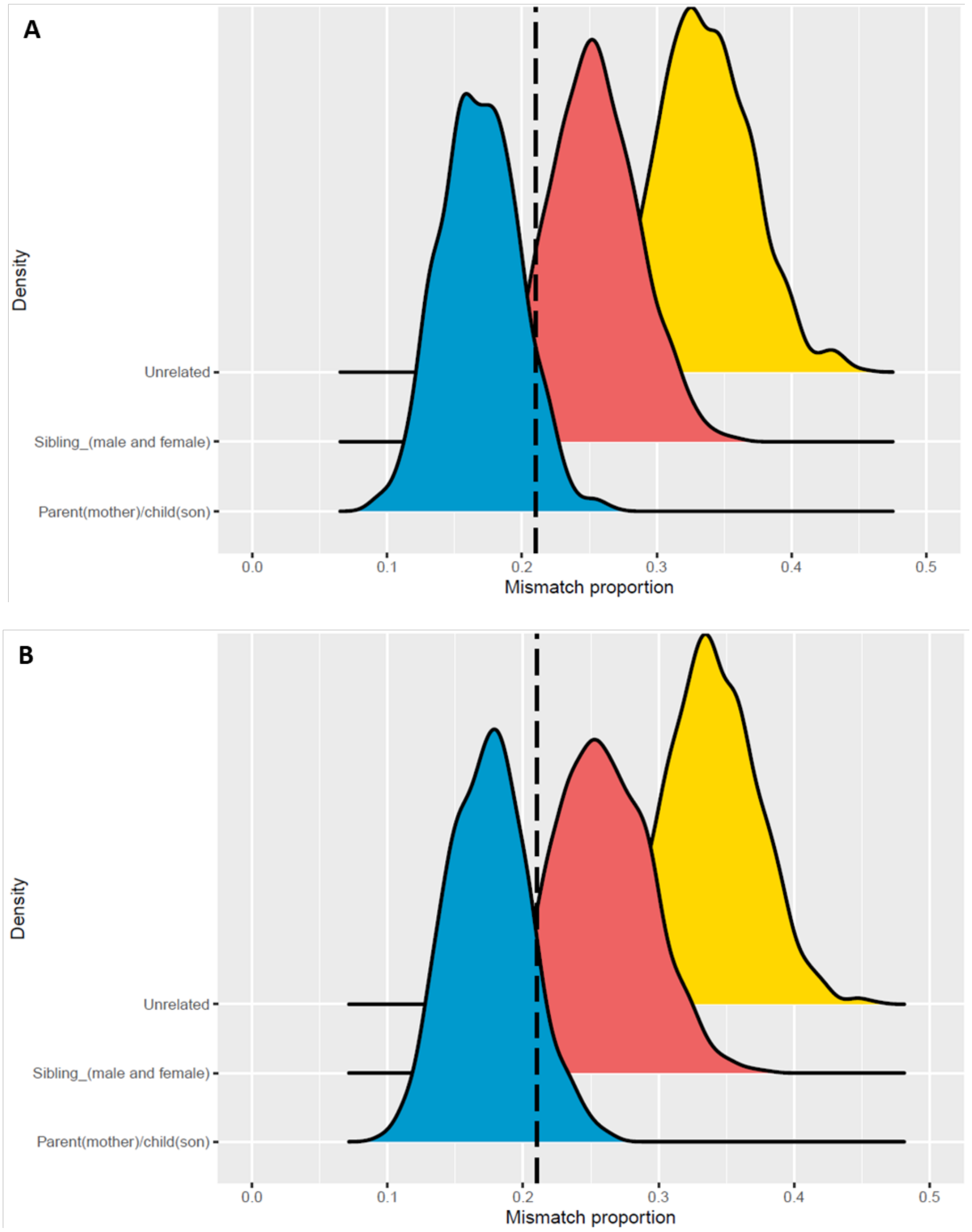

Figure S6. Observed mismatch proportions for burial 4 vs B comparisons (black dashed lines) in relation to the expected mismatch proportions (distribution plots) for various degrees of relatedness with (A) error rate= 0 and (B) error rate= 0.005. These plots are based on the analyses of X-chromosomal SNP data. Blue= parent (mother)/ child (son), red= sibling (male and female), yellow= unrelated (male and female), Related to Figure 4

Supplemental Data S2: Pedigree Simulations

Burials 3 and B Full Sibling Prediction

Simulated data, based on the Washington pedigree, for burials 3 (George Steptoe Washington Jr.) and B (Dr. Samuel Walter Washington) were aligned with data from the generic full siblings' relationship (Figure S7). Figure S7 demonstrates that full siblings share a greater number of long, chromosomal segments compared to half siblings (2nd degree relatives). No overlap was observed in the datapoints falling within the 99% confidence interval between the full and half sibling relationships, contrary to the more distant relationships (Figures S8-S10), suggesting that estimations for more closely related individuals are more reliable. Therefore, this simulation data supports the kinship predictions amongst the three burials calculated in the Parabon Fx software.

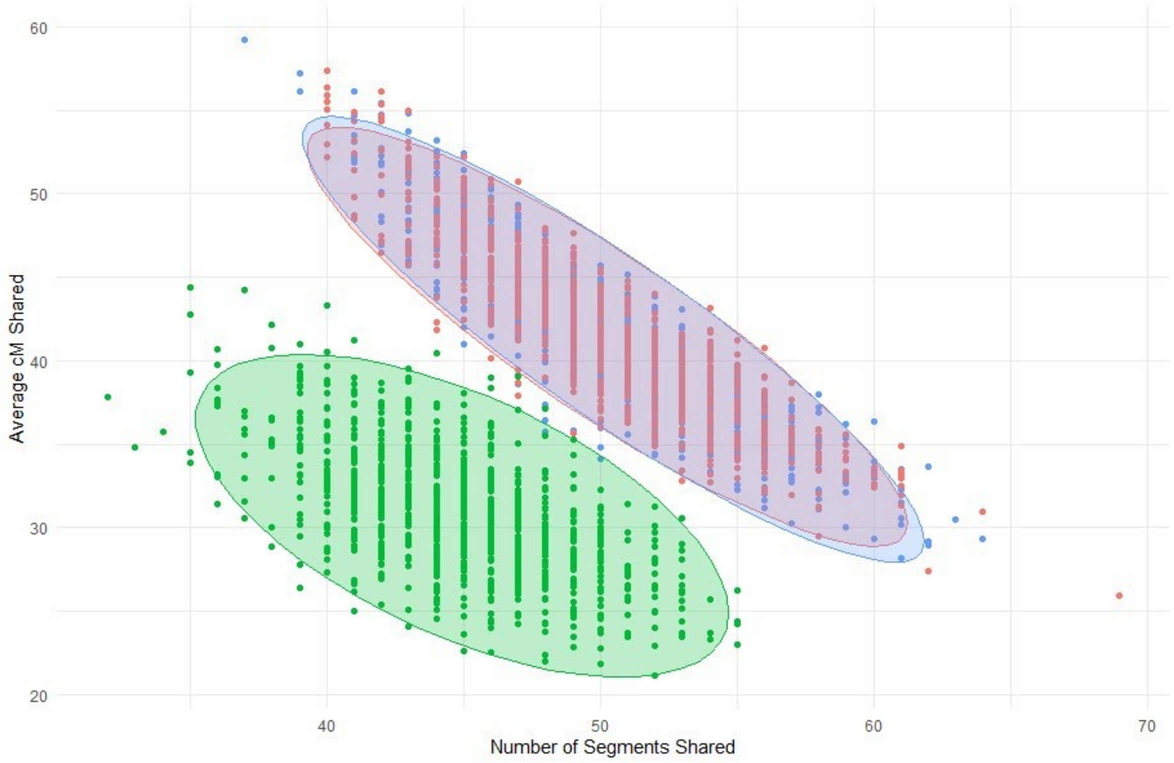

Figure S7. Data generated from 1000 simulations comparing the average centimorgans (cM) and number of segments shared between burials 3 and B (blue), 1st degree full siblings (red), and 2nd degree half siblings (green), Related to Figure 5

Burial 3 and S.W.W. 4th Degree Relative Prediction

Simulation data for burial 3 versus S.W.W., based on the Washington pedigree, overlapped with data for a great-great-uncle (4th degree) and a great-great-great-uncle (5th degree; Figure S8). Overlap between the 4th and 5th degree pedigree simulations, demonstrate that there is a wide range of DNA shared IBD at the more distant relationships and thus these relationships are not as easily resolved. Overall, the Washington pedigree simulations include more datapoints with a larger number of chromosomal segments shared between burial 3 and S.W.W., causing the ellipse to shift towards the closer, great-great-uncle (4th degree) relationship, which is consistent with the Parabon Fx results.

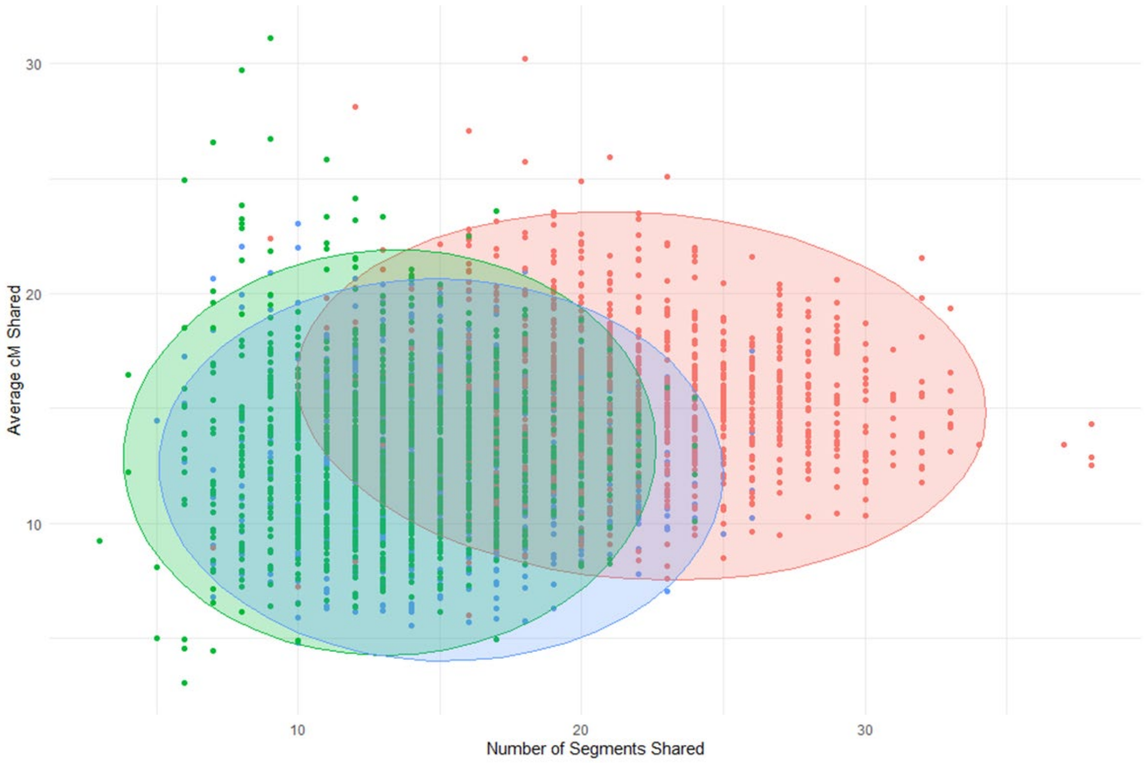

Figure S8. Data generated from 1000 simulations comparing the average centimorgans (cM) and number of segments shared between burial 3 and Samuel Walter Washington (blue), a 4th degree great-great uncle (red), and a 5th degree great-great-great uncle (green), Related to Figure 5

Burial B and S.W.W. 3rd Degree Relative Prediction

Simulation data for burial B versus S.W.W. also resulted in an overlap with data from pedigrees for a great-grandfather (3rd degree relative) and a great-great-grandfather (4th degree relative; Figure S9). The data further support the wide range of shared DNA observed in more distantly related individuals. Consistent with the observations made based on Figure S8, the Washington pedigree dataset includes more datapoints with a larger number of shared chromosomal segments between burial B and S.W.W. This causes the ellipse to shift towards a closer degree of relatedness (3rd degree relationship), than expected which is consistent with Parabon Fx prediction.

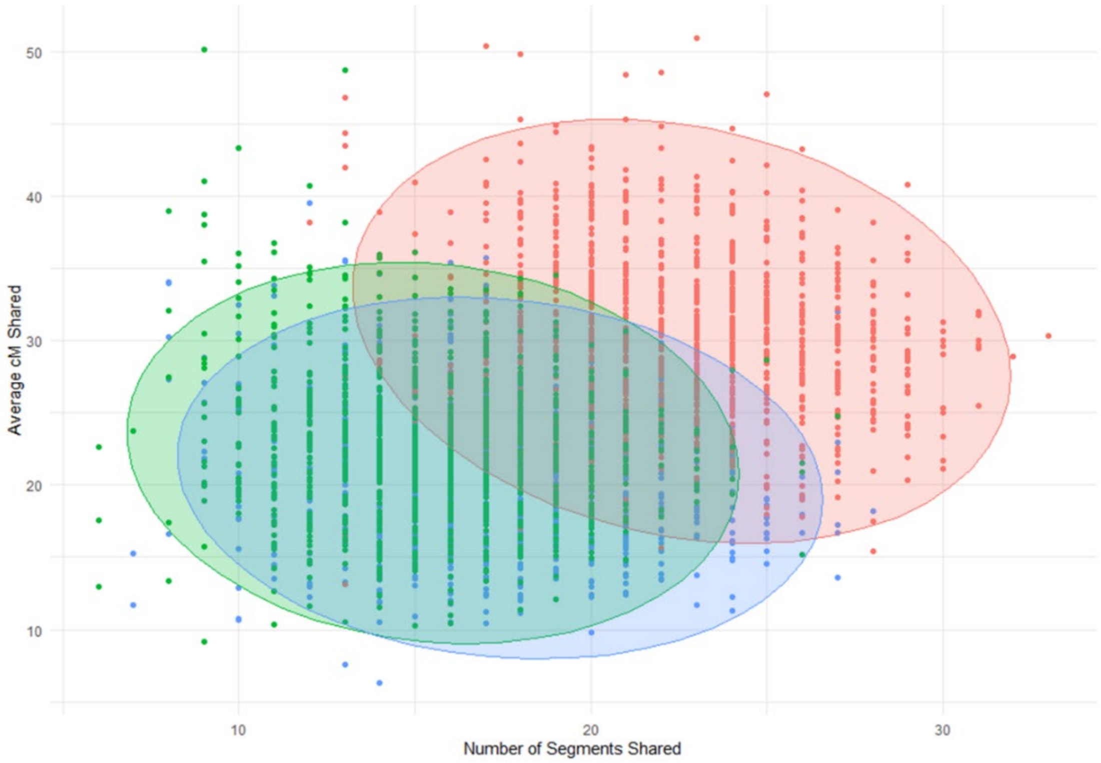

Figure S9. Data generated from 1000 simulations comparing the average centimorgans (cM) and number of segments shared between burial B and Samuel Walter Washington (blue), a 3rd degree great-grandparent (red), and a 4th degree great-great-grandparent (green), Related to Figure 5

Burial 4 and S.W.W. 4th Degree Relative Prediction

Simulation data for burial 4 versus S.W.W. resulted in an overlap with data from pedigrees for a great-great-grandmother (4th degree) and a great-great-great-grandmother (5th degree; Figure S10). The data demonstrates an even wider range of shared DNA between 4th and 5th degree relatives. The ellipse for the Washington pedigree (blue) aligns with the 5th degree relationship pedigree (green) and does not exhibit a shift towards the closer relationship. Although an additional shift was not observed, this does not indicate that the Parabon Fx prediction was incorrect, rather, that the limited Washington pedigree may be missing instances of pedigree collapse that directly impact Lucy Payne.

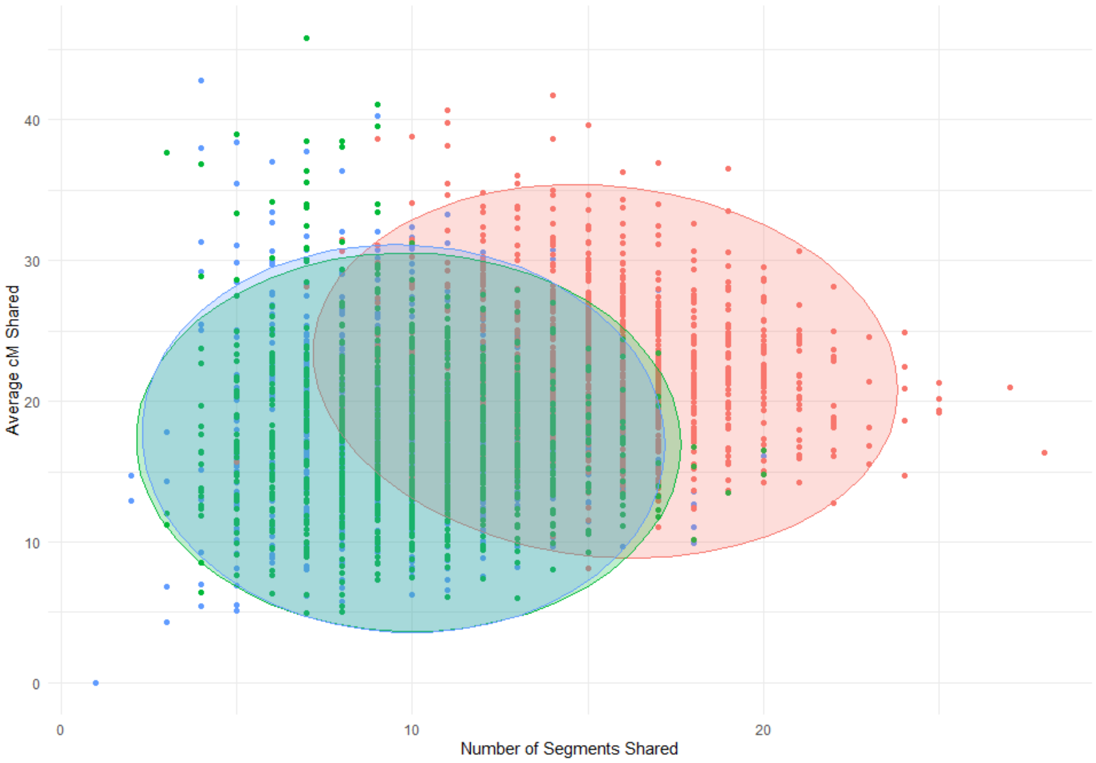

Figure S10. Data generated from 1000 simulations comparing the average centimorgans (cM) and number of segments shared between burial 4 and Samuel Walter Washington (blue), a 4th degree great-great-grandparent (red), and a 5th degree great-great-great-grandparent (green), Related to Figure 5
